# Waterborne Risperidone Decreases Stress Response in Zebrafish

**DOI:** 10.1371/journal.pone.0140800

**Published:** 2015-10-16

**Authors:** Renan Idalencio, Fabiana Kalichak, João Gabriel Santos Rosa, Tiago Acosta de Oliveira, Gessi Koakoski, Darlan Gusso, Murilo Sander de Abreu, Ana Cristina Varrone Giacomini, Heloísa Helena de Alcântara Barcellos, Angelo L. Piato, Leonardo José Gil Barcellos

**Affiliations:** 1 Programa de Pós-Graduação em Bioexperimentação, Universidade de Passo Fundo (UPF), Passo Fundo, RS, Brasil; 2 Laboratório de Fisiologia de Peixes, Universidade de Passo Fundo (UPF), Passo Fundo, RS, Brasil; 3 Programa de Pós-Graduação em Farmacologia, Universidade Federal de Santa Maria (UFSM), Santa Maria, RS, Brasil; 4 Laboratório de Psicofarmacologia e Comportamento (LAPCOM), Programa de Pós-Graduação em Farmacologia e Terapêutica, Universidade Federal do Rio Grande do Sul (UFRGS), Porto Alegre, RS, Brasil; Queen Mary University of London, UNITED KINGDOM

## Abstract

The presence of drugs and their metabolites in surface waters and municipal effluents has been reported in several studies, but its impacts on aquatic organisms are not yet well understood. This study investigated the effects of acute exposure to the antipsychotic risperidone on the stress and behavioral responses in zebrafish. It became clear that intermediate concentration of risperidone inhibited the hypothalamic-pituitary-interrenal axis and displayed anxiolytic-like effects in zebrafish. The data presented here suggest that the presence of this antipsychotic in aquatic environments can alter neuroendocrine and behavior profiles in zebrafish.

## Introduction

The contamination of water resources like natural water bodies or urban effluents by pharmaceutical drugs and/or its metabolites has been reported since the 1970’s, consequently increasing the concern with health consequences for human population as well as for the aquatic life [1–9]. Risperidone, an atypical antipsychotic, has high affinity for serotonin type 2 (5-HT_2_) and dopamine type 2 (D2) receptors. This drug is widely used for the treatment of schizophrenia and bipolar disorder. In people with schizophrenia in maintenance treatment, risperidone induces relatively few extrapyramidal syndromes (EPS), especially less akathisia and tremor, as compared to typical antipsychotic haloperidol. As expected, due to its high prescription rates and off-label uses [10–13], risperidone has been detected in urban water wastes [3].

The activation of the hypothalamus-pituitary-interrenal (HPI) axis and the consequent cortisol elevation is an important reaction of the organism against physical stressors, predator encounters, and chemical stressors such as drug contaminations, aiming to restore the homeostasis [14–18] with generalized effects in metabolism, growth, immune system and osmoregulation processes [19–21]. Thus, pollutants that interfere with the fish stress response can adversely affect their survival [22–23].

We hypothesized that risperidone residues, due its central effects, might alter the functioning of the stress neuroendocrine axis, impairing the general response of the fish residing in contaminated water bodies. We tested our hypothesis using zebrafish (*Danio rerio*) as the experimental model, since this species presents many advantages such as easy handling and maintenance as well as shows high genetic homology with humans [24–26]. Several recent studies have reinforced its use as an organism model for stress research [26–32].

## Materials and Methods

### Ethical note

This study followed the guidelines of Conselho Nacional de Controle de Experimentação Animal (CONCEA) and was approved by the Ethics Commission for Animal Use (CEUA) at Universidade de Passo Fundo, UPF, Passo Fundo, RS, Brazil (Protocol # 7/2013 –CEUA). The use of this method was justified to avoid potential confounding factors while interpreting data regarding the effects of risperidone on the stress response axis.

### Animals

A population of approximately 500 mixed-sex, adult wild-type zebrafish (*Danio rerio*) short-fin (SF) strain, weighing 0.7 to 0.9 g were held in glass aquaria with constant aeration and equipped with biological filtering under a natural photoperiod (approximately 14 h light: 10 h dark). Water was maintained at 26 ± 2°C and pH 7.0, with dissolved oxygen levels at 6 ± 0.5 mg/L, total ammonia levels at 0.01 mg/L, total hardness at 6 mg/L, and alkalinity at 22 mg/L CaCO_3_. Behavioral testing was performed during the afternoon.

### Experiment 1 – acute stress challenge

Zebrafish were distributed in 36 glass aquaria (30 x 30 x 30 cm, six fish per tank), acclimatized for seven days and fed with commercial food flakes (Tetra Min, Tetra, Melle, Germany). Twenty-four hours later, fish were exposed to risperidone for 15 minutes. Fish were then stressed by chasing them with a net for two minutes [28], and sampled after 0, 15, 60 and 240 minutes for whole-body cortisol analysis. Similarly, groups of fish were exposed to risperidone without stress (sampled at the same time points), aiming to evaluate an eventual stress effect of the risperidone *per se*. A basal situation, i.e. without drug exposure and stress test was performed as control. We used five risperidone concentrations (with or without stress) plus the controls (with or without stress). Thus, we have 12 glass aquaria with six fish each. We repeated this setup 3 times, using two fish from each aquaria in each replication, totalizing a final sample of 6 fish (n = 6).

Risperidone (Risperidon, 1 mg/mL, Laboratório EMS, Hortolândia, SP) was used in five concentrations: 0.00034 μg/L, 85 μg/L, 170 μg/L, 340 μg/L and 680 μg/L. The lower concentration was already detected in the environment [3] while the highest concentration was calculated based on the concentration that produced effect zebrafish behavior [33]. The environmental concentration was then multiplied by 250,000, 500,000, 1,000,000 and 2,000,000 to reach the intermediate concentrations of 85, 170, 340 and 680 μg/L, respectively.

### Experiment 2 – novel tank test

**Fig 1:**
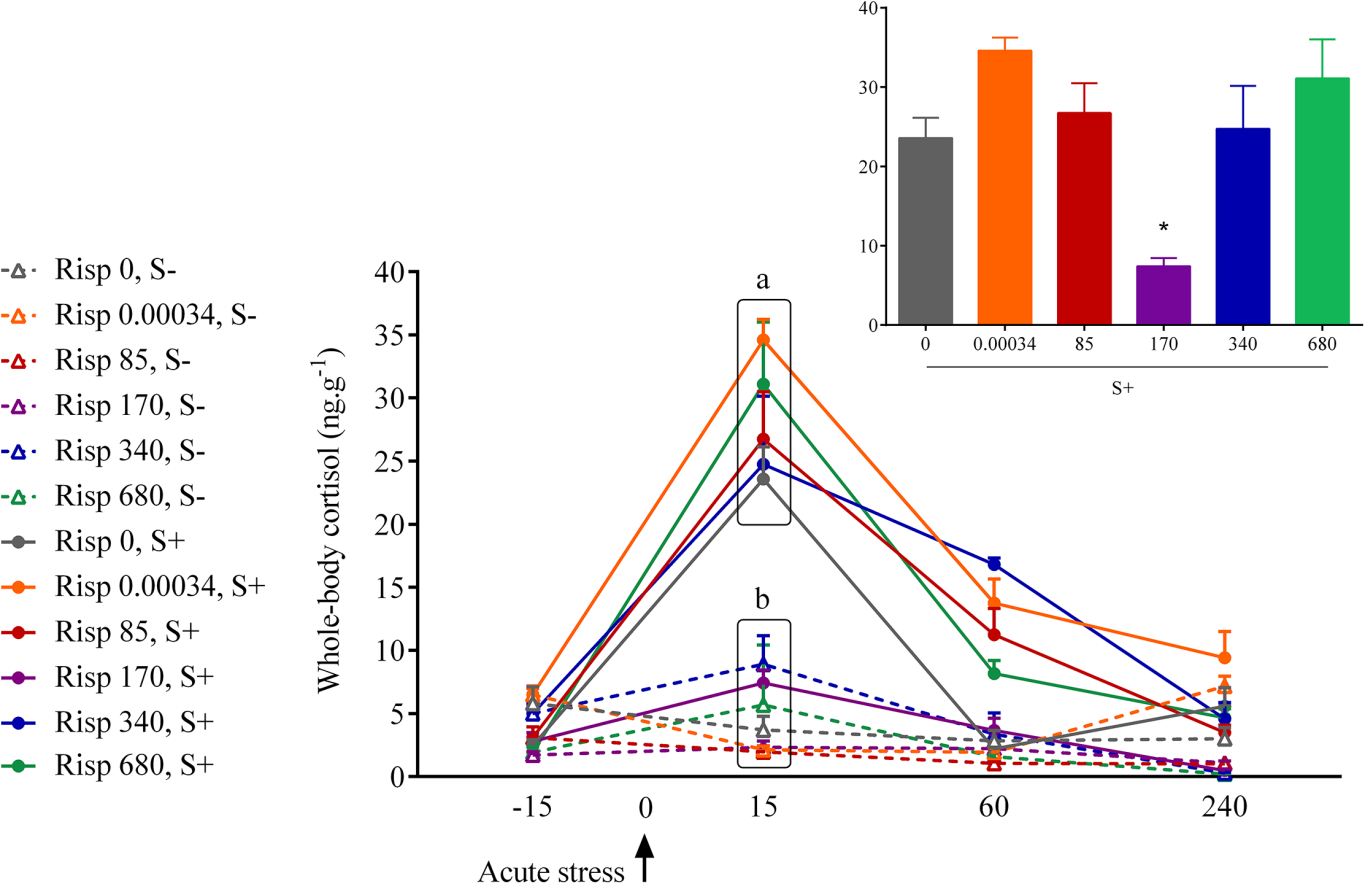
Whole-body cortisol concentrations in zebrafish exposed to risperidone followed by an acute stress test and respective controls. The values are expressed as the mean ± standard error of mean of 5–6 fish. Different small letters indicate significant group differences in each sampling time. The insert shows graphical demonstration of U-shaped dose response curve at the time of cortisol peak. S- and S+ refer to non-stressed and stressed fish, respectively.

We performed a behavioral test (novel tank test [26, 27]) using risperidone at the concentration of 170 μg/L, which impaired cortisol response in experiment 1 (see results section, Fig 1). For this purpose, 24 zebrafish not exposed in the experiment 1, were distributed in four groups: (1) control (no exposure to risperidone, no stress), (2) risperidone exposed, (3) stress and (4) risperidone exposed + stress. After 15 min of exposure, fish were individually transferred to the novel tank (4 x 20 x 20 cm) and video recorded for 5 minutes, 15 min after stressor application. The videos were then analyzed using AnyMaze® video tracking system (Stoelting, CO, USA) and the following parameters were analyzed: total distance (m), mean speed (m/s), absolute turn angle, number of crossings between different compartments of the tank (upper, middle and bottom) and the time spent in upper, middle and bottom compartments.

### Procedures–whole-body cortisol extraction and analysis

Fish were captured and immediately frozen in liquid nitrogen for 10–30 s, followed by storage at -20°C until cortisol extraction. Whole-body cortisol was extracted using the method described by Oliveira et al. [28]. Tissue extracts were resuspended in 1mL PBS, and whole-body cortisol levels were measured in duplicate for each extraction using a commercially available enzyme-linked immune sorbent as say kit (EIAgen CORTISOL test, Bio Chem Immuno Systems). This kit was fully validated for zebrafish tissue extracts using the methodology described by Sink et al. [34].

### Statistics

Whole-body cortisol levels were analyzed by three-way Analysis of Variance (ANOVA) with treatment, stress and sampling time as the independent variables, followed by two-way ANOVAs restricted to each stress condition. Differences at each time point were analyzed by Bonferroni post-hoc test. Behavioral parameters were analyzed by two-way ANOVAs with treatment and stress as the independent variables, followed by Tukey’s multiple range post-hoc test. For both data sets, the homogeneity of variance was determined using Hartley’s test, and normality was determined using the Kolmogorov-Smirnov test. Data were expressed as means ± standard error of mean (S.E.M). Significance level was set at p<0.05.

## Results

### Experiment 1 – acute stress challenge

The acute stress protocol increased cortisol levels in zebrafish. There was a significant interaction among treatment, stress and time. Zebrafish exposed to 170 mg/L of risperidone and submitted to the acute stress test had a reduced cortisol response to an acute stressor at 15, 60 and 240 minutes after stress (Fig 1). Significant effects were observed in the analysis restricted to non-stressed animals, but cortisol levels in these groups are within the normal range reported in the literature. Table 1 summarizes the results yielded by the statistical analysis. The cortisol raw data and the complete statistical analysis are presented in supporting information (S1 File and S2 File).

**Table 1:** Results of analysis of variance (ANOVA) for cortisol levels.

Analysis	Effects	F-value	DF	P-value
3-way ANOVA	Treatment	29.650	5,233	**0.0001**
	Stress	421.812	1,233	**0.0001**
	Time	165.849	3,233	**0.0001**
	Treatment × stress	16.413	5,233	**0.0001**
	Treatment × time	5.598	15,233	**0.0001**
	Stress × time	169.065	3,233	**0.0001**
	Treatment × stress × time	6.667	15,233	**0.0001**
2-way ANOVA: S-	Treatment	16.687	5,116	**0.0001**
	Time	10.144	3,116	**0.0001**
	Treatment × time	6.355	15,116	**0.0001**
2-way ANOVA: S+	Treatment	23.401	5,117	**0.0001**
	Time	188.867	3,117	**0.0001**
	Treatment × time	5.792	15,117	**0.0001**

The table summarizes the main effects of and the interaction between treatment, stress and time, as well as the restricted ANOVAs to each stress condition. DF refers to degrees of freedom. Significant effects (p<0.05) are given in bold font.

### Experiment 2 – novel tank test

**Fig 2:**
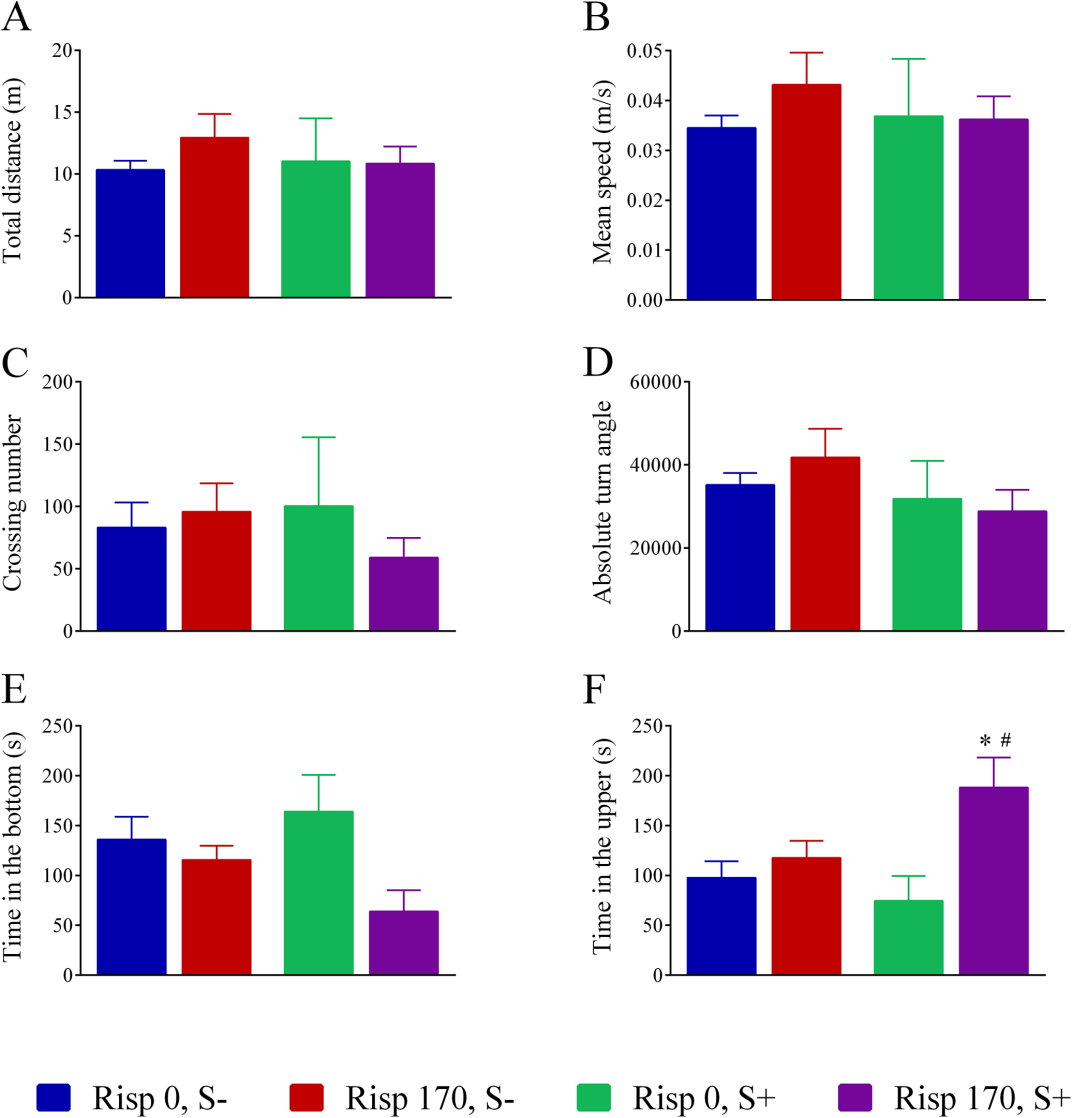
Behavioral parameters of zebrafish in the novel tank test followed by an acute stress protocol. Total distance (A), mean speed (B), crossings between compartments (C), absolute turn angle (D), time spent in the bottom (E), and time in the upper (F). The data are expressed as the mean ± standard error of mean of 5–6 fish. * = p<0.05 compared to Risp 0, S- group; ^#^ = p<0.05 compared to Rips 0, S+ group. S- and S+ refer to non-stressed and stressed fish, respectively.

The Fig 2 shows the effects of risperidone (170 μg/L) in the novel tank test. A significant main effect of treatment was observed for time in the bottom and upper, and a significant interaction between treatment and stress was observed for time in the upper. Table 2 summarizes the results yielded by the statistical analysis for each behavioral parameter evaluated. Post hoc analysis revealed that risperidone increased time spent in the upper in stressed fish. Total distance, mean speed, absolute turn angle, and number of crossings did not differ among experimental groups. The behavioral raw data and the complete statistical analysis are presented in supporting information (S3 File and S4 File).

**Table 2:** Results of analysis of variance (ANOVA) for the behavioral tests.

Dependent variable	Effects	F-value	DF	P-value
Total distance	Treatment	0.302	1,19	0.589
	Stress	0.103	1,19	0.751
	Treatment × stress	0.405	1,19	0.532
Crossings	Treatment	0.175	1,19	0.680
	Stress	0.084	1,19	0.775
	Treatment × stress	0.637	1,19	0.435
Mean speed	Treatment	0.300	1,19	0.591
	Stress	0.100	1,19	0.756
	Treatment × stress	0.401	1,19	0.534
Absolute turn angle	Treatment	0.074	1,19	0.788
	Stress	1.536	1,19	0.230
	Treatment × stress	0.530	1,19	0.476
Time in the bottom	Treatment	5.559	1,19	**0.029**
	Stress	0.212	1,19	0.650
	Treatment × stress	2.432	1,19	0.135
Time in the middle	Treatment	0.189	1,19	0.668
	Stress	0.661	1,19	0.426
	Treatment × stress	0.230	1,19	0.637
Time in the upper	Treatment	9.029	1,19	**0.007**
	Stress	1.145	1,19	0.298
	Treatment × stress	4.469	1,19	**0.048**

The table summarizes the main effects of and the interaction between treatment and stress. DF refers to degrees of freedom. Significant effects (p<0.05) are given in bold font.

## Discussion

Here we showed that acute exposure to an intermediate concentration of risperidone of 170 μg/L impaired the stress axis response, since the exposed zebrafish had lower cortisol levels than control fish, when exposed to an acute stress challenge. We also showed that zebrafish exposed to this concentration of risperidone decreased the time spent in the bottom compartment of the tank when compared with stressed fish, reinforcing the anxiolytic effect of this drug [35–38]. To our knowledge, this is the first report about risperidone effects on the stress neuroendocrine axis in zebrafish.

The risperidone mechanism is yet unclear, but it acts directly on the central nervous system, more specifically in dopaminergic and serotoninergic receptors, probably without any direct effect on interrenal tissue [13, 39]. The serotoninergic system plays an essential role in the fish stress response [40].

Considering the concentrations used, the HPI impairing effect was verified only in the intermediate concentration of 170 μg/L. The lowest (0.00034 μg/L and 85 μg/L) and highest (340 μg/L and 680 μg/L) concentrations did not block the cortisol elevation after the acute stress challenge. Thus, the dose-response curve presented a U-shaped response (see the insert in the Fig 1). This type of response was verified previously for the effects of diazepam [41] and ethanol [28] on stress response and behavior [42]. Similar U-shaped curve was also found for the cortisol effects on human memory [43].

The biological meaning of the minor cortisol fluctuations induced by risperidone in non-stressed animals remains to be elucidated. Nevertheless, the risperidone concentration that blunted the cortisol increase in stressed animals did not alter cortisol in non-stressed, control animals, suggesting that risperidone does not influence basal hormonal levels per se, but rather it modulates the response elicited by stress.

Regarding the novel tank test, zebrafish exposed to risperidone showed a decrease in the time spent in the tank bottom and stressed fish exposed to risperidone spent more time in the upper part of the tank, demonstrating its anxiolytic effects. This anxiolytic-like effect of atypical antipsychotics was also verified in mammals [44–45]. The risperidone effect on zebrafish behavior was previously verified using a light-dark test, where the drug decreased the number of crossing between light and dark compartments [33]. In this study, we analyzed the zebrafish behavior at 15 minutes after stress. Thus, we cannot discard that more clear behavioral effects of risperidone exposure could be detected if analyzed earlier than 15 minutes after stress exposure.

The anxiolytic effect of risperidone is not well understood since the exact mechanisms by which it blocks the HPI functioning in response to a stress challenge are still unclear. Despite the unclear mechanism, a fish with an impaired capacity to respond and cope with stress lost its ability to maintain homeostasis against stressors by reducing the ability to promote the necessary adjustments [22–23, 46–47]. Also, the behavioral changes produced by risperidone exposure may reduce the caution in predator inspection and consequently increase the risk of predation [48]. Combining the neuroendocrine and behavioral results and considering that these effects were detected in a concentration much higher than the one found in the environment, the consequences of an environmental contamination with risperidone are difficult to predict.

The concentration of 170 μg/L of risperidone is unexpected in natural environments. However, aquatic organisms may be exposed to accidental spills of pollutants, incorrect discharges of substances or contaminants already present in the water. Such contamination may cause biomagnification, in which concentrations much higher than those found in the environment may be observed. This phenomenon has been reported for the presence of pesticides [49–51].

Our results highlight that the presence of risperidone residues in aquatic ecosystems may blunt the cortisol response to stress as well the fish behavior with consequences on fish survival and welfare.

## Supporting Information

S1 File Idalencio et al_Cortisol data. Raw data of cortisol determination.
(PDF)

S2 File Idalencio et al_Cortisol results. Statistics of cortisol data.
(PDF)

S3 File Idalencio et al_Behavior data. Raw data of behavioral tests.
(PDF)

S4 File Idalencio et al_Behavior results. Statistics of behavioral data.
(PDF)
