# Supplementary material for: Waterborne Risperidone Decreases Stress Response in Zebrafish
Source: PLoS One. 2015 Oct 16;10(10):e0140800. doi: 10.1371/journal.pone.0140800 (PMC4608780; doi:10.1371/journal.pone.0140800)
Supplement: S1 File — Raw data of cortisol determination. (PDF) [file pone.0140800.s001.pdf]

	tto	stress	time	cortisol	groups
1	risp0	S-	t1	4,00000	R0S-
2	risp0	S-	t1	5,00000	R0S-
3	risp0	S-	t1	4,00000	R0S-
4	risp0	S-	t1	11,00000	R0S-
5	risp0	S-	t1	5,00000	R0S-
6	risp0.00032	S-	t1	8,00000	R00032S-
7	risp0.00032	S-	t1	8,00000	R00032S-
8	risp0.00032	S-	t1	5,90000	R00032S-
9	risp0.00032	S-	t1	5,30000	R00032S-
10	risp0.00032	S-	t1	3,90000	R00032S-
11	risp0.00032	S-	t1	7,80000	R00032S-
12	risp85	S-	t1	6,50000	R85S-
13	risp85	S-	t1	3,00000	R85S-
14	risp85	S-	t1	4,50000	R85S-
15	risp85	S-	t1	2,00000	R85S-
16	risp85	S-	t1	1,50000	R85S-
17	risp85	S-	t1	1,00000	R85S-
18	risp170	S-	t1	1,50000	R170S-
19	risp170	S-	t1	1,00000	R170S-
20	risp170	S-	t1	2,00000	R170S-
21	risp170	S-	t1	2,75000	R170S-
22	risp170	S-	t1	1,00000	R170S-
23	risp170	S-	t1	2,00000	R170S-
24	risp340	S-	t1	2,00000	R340S-
25	risp340	S-	t1	6,00000	R340S-
26	risp340	S-	t1	4,50000	R340S-
27	risp340	S-	t1	5,00000	R340S-
28	risp340	S-	t1	5,00000	R340S-
29	risp340	S-	t1	7,50000	R340S-
30	risp680	S-	t1	,75000	R680S-
31	risp680	S-	t1	2,00000	R680S-
32	risp680	S-	t1	1,25000	R680S-
33	risp680	S-	t1	4,00000	R680S-
34	risp680	S-	t1	2,00000	R680S-
35	risp680	S-	t1	1,50000	R680S-
36	risp0	S+	t1	3,00000	R0S+
37	risp0	S+	t1	3,25000	R0S+
38	risp0	S+	t1	1,00000	R0S+
39	risp0	S+	t1	1,50000	R0S+

	tto	stress	time	cortisol	groups
40	risp0	S+	t1	4,00000	R0S+
41	risp0	S+	t1	2,00000	R0S+
42	risp0.00032	S+	t1	8,00000	R00032S+
43	risp0.00032	S+	t1	7,50000	R00032S+
44	risp0.00032	S+	t1	5,00000	R00032S+
45	risp0.00032	S+	t1	6,50000	R00032S+
46	risp0.00032	S+	t1	5,50000	R00032S+
47	risp0.00032	S+	t1	6,50000	R00032S+
48	risp85	S+	t1	3,00000	R85S+
49	risp85	S+	t1	3,20000	R85S+
50	risp85	S+	t1	,50000	R85S+
51	risp85	S+	t1	5,20000	R85S+
52	risp85	S+	t1	1,20000	R85S+
53	risp85	S+	t1	5,50000	R85S+
54	risp170	S+	t1	4,75000	R170S+
55	risp170	S+	t1	4,50000	R170S+
56	risp170	S+	t1	1,00000	R170S+
57	risp170	S+	t1	2,00000	R170S+
58	risp170	S+	t1	1,00000	R170S+
59	risp170	S+	t1	3,50000	R170S+
60	risp340	S+	t1	3,30000	R340S+
61	risp340	S+	t1	6,70000	R340S+
62	risp340	S+	t1	3,80000	R340S+
63	risp340	S+	t1	6,20000	R340S+
64	risp340	S+	t1	3,00000	R340S+
65	risp340	S+	t1	7,00000	R340S+
66	risp680	S+	t1	1,90000	R680S+
67	risp680	S+	t1	1,00000	R680S+
68	risp680	S+	t1	3,30000	R680S+
69	risp680	S+	t1	,70000	R680S+
70	risp680	S+	t1	1,50000	R680S+
71	risp680	S+	t1	3,10000	R680S+
72	risp0	S-	t2	3,00000	R0S-
73	risp0	S-	t2	5,00000	R0S-
74	risp0	S-	t2	7,00000	R0S-
75	risp0	S-	t2	,50000	R0S-
76	risp0	S-	t2	3,00000	R0S-
77	risp0.00032	S-	t2	1,75000	R00032S-
78	risp0.00032	S-	t2	2,00000	R00032S-

	tto	stress	time	cortisol	groups
79	risp0.00032	S-	t2	2,00000	R00032S-
80	risp0.00032	S-	t2	2,00000	R00032S-
81	risp0.00032	S-	t2	1,50000	R00032S-
82	risp0.00032	S-	t2	3,50000	R00032S-
83	risp85	S-	t2	3,00000	R85S-
84	risp85	S-	t2	1,00000	R85S-
85	risp85	S-	t2	1,50000	R85S-
86	risp85	S-	t2	3,50000	R85S-
87	risp85	S-	t2	2,25000	R85S-
88	risp85	S-	t2	,50000	R85S-
89	risp170	S-	t2	2,00000	R170S-
90	risp170	S-	t2	1,50000	R170S-
91	risp170	S-	t2	2,00000	R170S-
92	risp170	S-	t2	3,75000	R170S-
93	risp170	S-	t2	3,75000	R170S-
94	risp170	S-	t2	1,00000	R170S-
95	risp340	S-	t2	6,50000	R340S-
96	risp340	S-	t2	6,00000	R340S-
97	risp340	S-	t2	6,00000	R340S-
98	risp340	S-	t2	6,00000	R340S-
99	risp340	S-	t2	7,50000	R340S-
100	risp340	S-	t2	8,00000	R340S-
101	risp680	S-	t2	1,00000	R680S-
102	risp680	S-	t2	,37500	R680S-
103	risp680	S-	t2	1,50000	R680S-
104	risp680	S-	t2	1,00000	R680S-
105	risp680	S-	t2	,15000	R680S-
106	risp680	S-	t2	1,50000	R680S-
107	risp0	S+	t2	18,00000	R0S+
108	risp0	S+	t2	25,50000	R0S+
109	risp0	S+	t2	35,00000	R0S+
110	risp0	S+	t2	19,00000	R0S+
111	risp0	S+	t2	23,00000	R0S+
112	risp0	S+	t2	21,00000	R0S+
113	risp0.00032	S+	t2	32,50000	R00032S+
114	risp0.00032	S+	t2	30,00000	R00032S+
115	risp0.00032	S+	t2	38,00000	R00032S+
116	risp0.00032	S+	t2	38,50000	R00032S+
117	risp0.00032	S+	t2	34,00000	R00032S+

	tto	stress	time	cortisol	groups
118	risp85	S+	t2	38,00000	R85S+
119	risp85	S+	t2	21,50000	R85S+
120	risp85	S+	t2	14,50000	R85S+
121	risp85	S+	t2	26,00000	R85S+
122	risp85	S+	t2	37,00000	R85S+
123	risp85	S+	t2	23,50000	R85S+
124	risp170	S+	t2	6,50000	R170S+
125	risp170	S+	t2	4,50000	R170S+
126	risp170	S+	t2	7,00000	R170S+
127	risp170	S+	t2	12,00000	R170S+
128	risp170	S+	t2	8,00000	R170S+
129	risp340	S+	t2	20,00000	R340S+
130	risp340	S+	t2	32,00000	R340S+
131	risp340	S+	t2	21,00000	R340S+
132	risp340	S+	t2	38,50000	R340S+
133	risp340	S+	t2	32,00000	R340S+
134	risp340	S+	t2	24,00000	R340S+
135	risp680	S+	t2	29,50000	R680S+
136	risp680	S+	t2	39,00000	R680S+
137	risp680	S+	t2	13,50000	R680S+
138	risp680	S+	t2	41,50000	R680S+
139	risp680	S+	t2	29,50000	R680S+
140	risp680	S+	t2	32,00000	R680S+
141	risp0	S-	t3	3,00000	R0S-
142	risp0	S-	t3	3,00000	R0S-
143	risp0	S-	t3	5,00000	R0S-
144	risp0	S-	t3	,05000	R0S-
145	risp0	S-	t3	1,00000	R0S-
146	risp0	S-	t3	5,00000	R0S-
147	risp0.00032	S-	t3	2,00000	R00032S-
148	risp0.00032	S-	t3	1,00000	R00032S-
149	risp0.00032	S-	t3	1,00000	R00032S-
150	risp0.00032	S-	t3	2,00000	R00032S-
151	risp0.00032	S-	t3	3,50000	R00032S-
152	risp0.00032	S-	t3	2,00000	R00032S-
153	risp85	S-	t3	1,50000	R85S-
154	risp85	S-	t3	1,00000	R85S-
155	risp85	S-	t3	1,25000	R85S-
156	risp85	S-	t3	,50000	R85S-

	tto	stress	time	cortisol	groups
157	risp85	S-	t3	1,00000	R85S-
158	risp85	S-	t3	1,00000	R85S-
159	risp170	S-	t3	2,00000	R170S-
160	risp170	S-	t3	2,50000	R170S-
161	risp170	S-	t3	2,25000	R170S-
162	risp170	S-	t3	2,50000	R170S-
163	risp170	S-	t3	2,00000	R170S-
164	risp170	S-	t3	2,00000	R170S-
165	risp340	S-	t3	1,00000	R340S-
166	risp340	S-	t3	1,50000	R340S-
167	risp340	S-	t3	11,00000	R340S-
168	risp340	S-	t3	1,50000	R340S-
169	risp340	S-	t3	5,00000	R340S-
170	risp340	S-	t3	,25000	R340S-
171	risp680	S-	t3	1,25000	R680S-
172	risp680	S-	t3	1,00000	R680S-
173	risp680	S-	t3	,15000	R680S-
174	risp680	S-	t3	1,50000	R680S-
175	risp680	S-	t3	5,50000	R680S-
176	risp680	S-	t3	,05000	R680S-
177	risp0	S+	t3	1,50000	R0S+
178	risp0	S+	t3	4,50000	R0S+
179	risp0	S+	t3	1,50000	R0S+
180	risp0	S+	t3	2,00000	R0S+
181	risp0	S+	t3	1,50000	R0S+
182	risp0.00032	S+	t3	7,00000	R00032S+
183	risp0.00032	S+	t3	16,00000	R00032S+
184	risp0.00032	S+	t3	10,00000	R00032S+
185	risp0.00032	S+	t3	13,50000	R00032S+
186	risp0.00032	S+	t3	16,00000	R00032S+
187	risp0.00032	S+	t3	20,00000	R00032S+
188	risp85	S+	t3	16,50000	R85S+
189	risp85	S+	t3	14,00000	R85S+
190	risp85	S+	t3	4,00000	R85S+
191	risp85	S+	t3	16,00000	R85S+
192	risp85	S+	t3	10,00000	R85S+
193	risp85	S+	t3	7,00000	R85S+
194	risp170	S+	t3	1,50000	R170S+
195	risp170	S+	t3	3,00000	R170S+

	tto	stress	time	cortisol	groups
196	risp170	S+	t3	6,00000	R170S+
197	risp170	S+	t3	3,00000	R170S+
198	risp170	S+	t3	7,00000	R170S+
199	risp170	S+	t3	1,50000	R170S+
200	risp340	S+	t3	17,50000	R340S+
201	risp340	S+	t3	16,00000	R340S+
202	risp340	S+	t3	16,00000	R340S+
203	risp340	S+	t3	18,50000	R340S+
204	risp340	S+	t3	16,00000	R340S+
205	risp680	S+	t3	7,00000	R680S+
206	risp680	S+	t3	11,50000	R680S+
207	risp680	S+	t3	8,00000	R680S+
208	risp680	S+	t3	6,00000	R680S+
209	risp680	S+	t3	11,00000	R680S+
210	risp680	S+	t3	5,50000	R680S+
211	risp0	S-	t4	5,00000	R0S-
212	risp0	S-	t4	1,00000	R0S-
213	risp0	S-	t4	1,00000	R0S-
214	risp0	S-	t4	5,00000	R0S-
215	risp0	S-	t4	1,00000	R0S-
216	risp0	S-	t4	5,00000	R0S-
217	risp0.00032	S-	t4	6,50000	R00032S-
218	risp0.00032	S-	t4	5,00000	R00032S-
219	risp0.00032	S-	t4	10,00000	R00032S-
220	risp0.00032	S-	t4	6,00000	R00032S-
221	risp0.00032	S-	t4	9,00000	R00032S-
222	risp0.00032	S-	t4	6,50000	R00032S-
223	risp85	S-	t4	1,00000	R85S-
224	risp85	S-	t4	1,00000	R85S-
225	risp85	S-	t4	1,00000	R85S-
226	risp85	S-	t4	1,00000	R85S-
227	risp85	S-	t4	1,01000	R85S-
228	risp170	S-	t4	1,50000	R170S-
229	risp170	S-	t4	1,00000	R170S-
230	risp170	S-	t4	,50000	R170S-
231	risp170	S-	t4	1,50000	R170S-
232	risp170	S-	t4	1,00000	R170S-
233	risp170	S-	t4	1,00000	R170S-
234	risp340	S-	t4	,37500	R340S-

	tto	stress	time	cortisol	groups
235	risp340	S-	t4	,25000	R340S-
236	risp340	S-	t4	,50000	R340S-
237	risp340	S-	t4	,25000	R340S-
238	risp340	S-	t4	,05000	R340S-
239	risp340	S-	t4	,25000	R340S-
240	risp680	S-	t4	,25000	R680S-
241	risp680	S-	t4	,25000	R680S-
242	risp680	S-	t4	,05000	R680S-
243	risp680	S-	t4	,25000	R680S-
244	risp680	S-	t4	,25000	R680S-
245	risp680	S-	t4	,15000	R680S-
246	risp0	S+	t4	5,00000	R0S+
247	risp0	S+	t4	3,00000	R0S+
248	risp0	S+	t4	10,00000	R0S+
249	risp0	S+	t4	3,50000	R0S+
250	risp0	S+	t4	2,00000	R0S+
251	risp0	S+	t4	10,00000	R0S+
252	risp0.00032	S+	t4	6,00000	R00032S+
253	risp0.00032	S+	t4	9,00000	R00032S+
254	risp0.00032	S+	t4	18,50000	R00032S+
255	risp0.00032	S+	t4	7,00000	R00032S+
256	risp0.00032	S+	t4	11,50000	R00032S+
257	risp0.00032	S+	t4	4,50000	R00032S+
258	risp85	S+	t4	2,00000	R85S+
259	risp85	S+	t4	6,00000	R85S+
260	risp85	S+	t4	3,50000	R85S+
261	risp85	S+	t4	4,00000	R85S+
262	risp85	S+	t4	2,50000	R85S+
263	risp85	S+	t4	3,00000	R85S+
264	risp170	S+	t4	1,00000	R170S+
265	risp170	S+	t4	,50000	R170S+
266	risp170	S+	t4	,25000	R170S+
267	risp170	S+	t4	,15000	R170S+
268	risp170	S+	t4	,50000	R170S+
269	risp170	S+	t4	,50000	R170S+
270	risp340	S+	t4	5,00000	R340S+
271	risp340	S+	t4	5,00000	R340S+
272	risp340	S+	t4	4,00000	R340S+
273	risp340	S+	t4	10,00000	R340S+

Dados renan cortisol.sav

	tto	stress	time	cortisol	groups
274	risp340	S+	t4	3,00000	R340S+
275	risp340	S+	t4	,50000	R340S+
276	risp680	S+	t4	3,50000	R680S+
277	risp680	S+	t4	6,50000	R680S+
278	risp680	S+	t4	2,00000	R680S+
279	risp680	S+	t4	6,50000	R680S+
280	risp680	S+	t4	4,50000	R680S+
281	risp680	S+	t4	5,00000	R680S+
