# Supplementary material for: Waterborne Risperidone Decreases Stress Response in Zebrafish
Source: PLoS One. 2015 Oct 16;10(10):e0140800. doi: 10.1371/journal.pone.0140800 (PMC4608780; doi:10.1371/journal.pone.0140800)
Supplement: S2 File — Statistics of cortisol data. (PDF) [file pone.0140800.s002.pdf]

```
UNIANOVA cortisol BY tto stress time
  /METHOD=SSTYPE(3)
  /INTERCEPT=INCLUDE
  /EMMEANS=TABLES(OVERALL)
  /PRINT=DESCRIPTIVE
  /CRITERIA=ALPHA(.05)
  /DESIGN=tto stress time tto*stress tto*time stress*time tto*stress*time.
```

Univariate Analysis of Variance

Between-Subjects Factors

		Value Label	N
tto	risp0	risp0	45
	risp0.00032	risp0.00032	47
	risp170	risp170	47
	risp340	risp340	47
	risp680	risp680	48
	risp85	risp85	47
stress	S-	S-	141
	S+	S+	140
time	t1	t1	71
	t2	t2	69
	t3	t3	70
	t4	t4	71

Descriptive Statistics

Dependent Variable: cortisol

tto	stress	time	Mean	Std. Deviation	N
risp0	S-	t1	5,8000000	2,94957624	5
		t2	3,7000000	2,43926218	5
		t3	2,8416667	2,02741625	6
		t4	3,0000000	2,19089023	6
		Total	3,7522727	2,51189917	22
	S+	t1	2,4583333	1,14473432	6
		t2	23,5833333	6,21624217	6
		t3	2,2000000	1,30384048	5
		t4	5,5833333	3,55551215	6
		Total	8,7282609	9,77500088	23
	Total	t1	3,9772727	2,67967942	11
		t2	14,5454545	11,38080521	11
		t3	2,5500000	1,68745370	11
		t4	4,2916667	3,12219571	12
		Total	6,2955556	7,55740773	45
risp0.00032	S-	t1	6,4833333	1,71745936	6
		t2	2,1250000	,70267347	6
		t3	1,9166667	,91742393	6
		t4	7,1666667	1,91485422	6
		Total	4,4229167	2,79572574	24
	S+	t1	6,5000000	1,14017543	6
		t2	34,6000000	3,62973828	5
		t3	13,7500000	4,66636904	6
		t4	9,4166667	5,07362461	6
		Total	15,2608696	11,37368331	23
	Total	t1	6,4916667	1,38987137	12
		t2	16,8863636	17,12139146	11
		t3	7,8333333	6,96201817	12
		t4	8,2916667	3,84032630	12
		Total	9,7265957	9,78602515	47
risp170	S-	t1	1,7083333	,67854010	6
		t2	2,3333333	1,15830336	6
		t3	2,2083333	,24579802	6
		t4	1,0833333	,37638633	6
		Total	1,8333333	,82970223	24
	S+	t1	2,7916667	1,69127664	6
		t2	7,6000000	2,77037904	5
		t3	3,6666667	2,31660671	6
		t4	,4833333	,29439203	6
		Total	3,4630435	3,11933914	23

Descriptive Statistics

Dependent Variable: cortisol

tto	stress	time	Mean	Std. Deviation	N
	Total	t1	2,2500000	1,35260691	12
		t2	4,7272727	3,36239228	11
		t3	2,9375000	1,74553001	12
		t4	,7833333	,44941039	12
		Total	2,6308511	2,38242097	47
risp340	S-	t1	5,0000000	1,81659021	6
		t2	6,6666667	,87559504	6
		t3	3,3750000	4,07967523	6
		t4	,2791667	,15034682	6
		Total	3,8302083	3,21038548	24
	S+	t1	5,0000000	1,82537667	6
		t2	27,9166667	7,36489421	6
		t3	16,8000000	1,15108644	5
		t4	4,5833333	3,13714307	6
		Total	13,4347826	10,77520760	23
	Total	t1	5,0000000	1,73624464	12
		t2	17,2916667	12,17198410	12
		t3	9,4772727	7,61614613	11
		t4	2,4312500	3,08808423	12
		Total	8,5303191	9,17793303	47
risp680	S-	t1	1,9166667	1,12546287	6
		t2	,9208333	,56134140	6
		t3	1,5750000	2,01016169	6
		t4	,2000000	,08366600	6
		Total	1,1531250	1,29359443	24
	S+	t1	1,9166667	1,07780641	6
		t2	30,8333333	9,85731539	6
		t3	8,1666667	2,54296415	6
		t4	4,6666667	1,75119007	6
		Total	11,3958333	12,64840527	24
	Total	t1	1,9166667	1,05061310	12
		t2	15,8770833	16,98042141	12
		t3	4,8708333	4,07751363	12
		t4	2,4333333	2,61502071	12
		Total	6,2744792	10,29049795	48
risp85	S-	t1	3,0833333	2,08366664	6
		t2	1,9583333	1,16636901	6
		t3	1,0416667	,33229003	6
		t4	1,0020000	,00447214	5
		Total	1,8047826	1,44172520	23

Descriptive Statistics

Dependent Variable: cortisol

tto	stress	time	Mean	Std. Deviation	N
	S+	t1	3,1000000	2,02780670	6
		t2	26,7500000	9,16924206	6
		t3	11,2500000	5,09656747	6
		t4	3,5000000	1,41421356	6
		Total	11,1500000	10,99600718	24
	Total	t1	3,0916667	1,96026823	12
		t2	14,3541667	14,36870745	12
		t3	6,1458333	6,34649002	12
		t4	2,3645455	1,64372360	11
		Total	6,5768085	9,15139312	47
Total	S-	t1	3,9471429	2,53266724	35
		t2	2,9292857	2,22180443	35
		t3	2,1597222	2,07439559	36
		t4	2,1538571	2,74267583	35
		Total	2,7929787	2,49041147	141
	S+	t1	3,6277778	2,15683116	36
		t2	25,4558824	10,59900485	34
		t3	9,2941176	5,98750928	34
		t4	4,7055556	3,85621535	36
		Total	10,5821429	10,77433513	140
	Total	t1	3,7852113	2,33824407	71
		t2	14,0293478	13,62666811	69
		t3	5,6250000	5,67690943	70
		t4	3,4476761	3,56925631	71
		Total	6,6737011	8,71500871	281

Tests of Between-Subjects Effects

Dependent Variable: cortisol

Source	Type III Sum of Squares	df	Mean Square	F	Sig.
Corrected Model	18851,263[a]	47	401,091	38,695	,000
Intercept	12789,662	1	12789,662	1233,888	,000
tto	1536,654	5	307,331	29,650	,000
stress	4372,224	1	4372,224	421,812	,000
time	5157,233	3	1719,078	165,849	,000
tto * stress	850,634	5	170,127	16,413	,000
tto * time	870,324	15	58,022	5,598	,000
stress * time	5257,235	3	1752,412	169,065	,000
tto * stress * time	1038,087	15	69,206	6,677	,000
Error	2415,123	233	10,365		
Total	33781,644	281			
Corrected Total	21266,386	280			

a. R Squared = ,886 (Adjusted R Squared = ,864)

Estimated Marginal Means

Grand Mean

Dependent Variable: cortisol

Mean	Std. Error	95% Confidence Interval	
		Lower Bound	Upper Bound
6,760	,192	6,381	7,140

```
SORT CASES  BY stress.
SPLIT FILE SEPARATE BY stress.
UNIANOVA cortisol BY stress time tto
  /METHOD=SSTYPE(3)
  /INTERCEPT=INCLUDE
  /EMMEANS=TABLES(OVERALL)
  /PRINT=DESCRIPTIVE
  /CRITERIA=ALPHA(.05)
  /DESIGN=stress time tto stress*time stress*tto time*tto stress*time*tto.
```

Univariate Analysis of Variance

stress = S-

Between-Subjects Factors[a]

		Value Label	N
stress	S-	S-	141
time	t1	t1	35
	t2	t2	35
	t3	t3	36
	t4	t4	35
tto	risp0	risp0	22
	risp0.00032	risp0.00032	24
	risp170	risp170	24
	risp340	risp340	24
	risp680	risp680	24
	risp85	risp85	23

a. stress = S-

Descriptive Statistics[a]

Dependent Variable: cortisol

stress	time	tto	Mean	Std. Deviation	N
S-	t1	risp0	5,8000000	2,94957624	5
		risp0.00032	6,4833333	1,71745936	6
		risp170	1,7083333	,67854010	6
		risp340	5,0000000	1,81659021	6
		risp680	1,9166667	1,12546287	6
		risp85	3,0833333	2,08366664	6
		Total	3,9471429	2,53266724	35
	t2	risp0	3,7000000	2,43926218	5
		risp0.00032	2,1250000	,70267347	6
		risp170	2,3333333	1,15830336	6
		risp340	6,6666667	,87559504	6
		risp680	,9208333	,56134140	6
		risp85	1,9583333	1,16636901	6
		Total	2,9292857	2,22180443	35
	t3	risp0	2,8416667	2,02741625	6
		risp0.00032	1,9166667	,91742393	6
		risp170	2,2083333	,24579802	6
		risp340	3,3750000	4,07967523	6
		risp680	1,5750000	2,01016169	6
		risp85	1,0416667	,33229003	6
		Total	2,1597222	2,07439559	36
	t4	risp0	3,0000000	2,19089023	6
		risp0.00032	7,1666667	1,91485422	6
		risp170	1,0833333	,37638633	6
		risp340	,2791667	,15034682	6
		risp680	,2000000	,08366600	6

Descriptive Statistics[a]

Dependent Variable: cortisol

stress	time	tto	Mean	Std. Deviation	N
		risp85	1,0020000	,00447214	5
		Total	2,1538571	2,74267583	35
	Total	risp0	3,7522727	2,51189917	22
		risp0.00032	4,4229167	2,79572574	24
		risp170	1,8333333	,82970223	24
		risp340	3,8302083	3,21038548	24
		risp680	1,1531250	1,29359443	24
		risp85	1,8047826	1,44172520	23
		Total	2,7929787	2,49041147	141
Total	t1	risp0	5,8000000	2,94957624	5
		risp0.00032	6,4833333	1,71745936	6
		risp170	1,7083333	,67854010	6
		risp340	5,0000000	1,81659021	6
		risp680	1,9166667	1,12546287	6
		risp85	3,0833333	2,08366664	6
		Total	3,9471429	2,53266724	35
	t2	risp0	3,7000000	2,43926218	5
		risp0.00032	2,1250000	,70267347	6
		risp170	2,3333333	1,15830336	6
		risp340	6,6666667	,87559504	6
		risp680	,9208333	,56134140	6
		risp85	1,9583333	1,16636901	6
		Total	2,9292857	2,22180443	35
	t3	risp0	2,8416667	2,02741625	6
		risp0.00032	1,9166667	,91742393	6
		risp170	2,2083333	,24579802	6
		risp340	3,3750000	4,07967523	6
		risp680	1,5750000	2,01016169	6
		risp85	1,0416667	,33229003	6
		Total	2,1597222	2,07439559	36
	t4	risp0	3,0000000	2,19089023	6
		risp0.00032	7,1666667	1,91485422	6
		risp170	1,0833333	,37638633	6
		risp340	,2791667	,15034682	6
		risp680	,2000000	,08366600	6
		risp85	1,0020000	,00447214	5
		Total	2,1538571	2,74267583	35
	Total	risp0	3,7522727	2,51189917	22
		risp0.00032	4,4229167	2,79572574	24
		risp170	1,8333333	,82970223	24
		risp340	3,8302083	3,21038548	24

Descriptive Statistics[a]

Dependent Variable: cortisol

stress	time	tto	Mean	Std. Deviation	N
		risp680	1,1531250	1,29359443	24
		risp85	1,8047826	1,44172520	23
		Total	2,7929787	2,49041147	141

a. stress = S-

Tests of Between-Subjects Effects[a]

Dependent Variable: cortisol

Source	Type III Sum of Squares	df	Mean Square	F	Sig.
Corrected Model	554,494[b]	23	24,108	8,989	,000
Intercept	1107,508	1	1107,508	412,924	,000
stress	,000	0	.	.	.
time	81,622	3	27,207	10,144	,000
tto	223,781	5	44,756	16,687	,000
stress * time	,000	0	.	.	.
stress * tto	,000	0	.	.	.
time * tto	255,658	15	17,044	6,355	,000
stress * time * tto	,000	0	.	.	.
Error	313,807	117	2,682		
Total	1968,204	141			
Corrected Total	868,301	140			

a. stress = S-

b. R Squared = ,639 (Adjusted R Squared = ,568)

Estimated Marginal Means

Grand Mean[a]

Dependent Variable: cortisol

Mean	Std. Error	95% Confidence Interval	
		Lower Bound	Upper Bound
2,808	,138	2,534	3,081

a. stress = S-

stress = S+

Between-Subjects Factors[a]

		Value Label	N
stress	S+	S+	140
time	t1	t1	36
	t2	t2	34
	t3	t3	34
	t4	t4	36
tto	risp0	risp0	23
	risp0.00032	risp0.00032	23
	risp170	risp170	23
	risp340	risp340	23
	risp680	risp680	24
	risp85	risp85	24

a. stress = S+

Descriptive Statistics[a]

Dependent Variable: cortisol

stress	time	tto	Mean	Std. Deviation	N
S+	t1	risp0	2,4583333	1,14473432	6
		risp0.00032	6,5000000	1,14017543	6
		risp170	2,7916667	1,69127664	6
		risp340	5,0000000	1,82537667	6
		risp680	1,9166667	1,07780641	6
		risp85	3,1000000	2,02780670	6
		Total	3,6277778	2,15683116	36
	t2	risp0	23,5833333	6,21624217	6
		risp0.00032	34,6000000	3,62973828	5
		risp170	7,6000000	2,77037904	5
		risp340	27,9166667	7,36489421	6
		risp680	30,8333333	9,85731539	6
		risp85	26,7500000	9,16924206	6
		Total	25,4558824	10,59900485	34
	t3	risp0	2,2000000	1,30384048	5
		risp0.00032	13,7500000	4,66636904	6
		risp170	3,6666667	2,31660671	6
		risp340	16,8000000	1,15108644	5
		risp680	8,1666667	2,54296415	6
		risp85	11,2500000	5,09656747	6
		Total	9,2941176	5,98750928	34
	t4	risp0	5,5833333	3,55551215	6
		risp0.00032	9,4166667	5,07362461	6
		risp170	,4833333	,29439203	6
		risp340	4,5833333	3,13714307	6
		risp680	4,6666667	1,75119007	6

Descriptive Statistics[a]

Dependent Variable: cortisol

stress	time	tto	Mean	Std. Deviation	N
		risp85	3,5000000	1,41421356	6
		Total	4,7055556	3,85621535	36
	Total	risp0	8,7282609	9,77500088	23
		risp0.00032	15,2608696	11,37368331	23
		risp170	3,4630435	3,11933914	23
		risp340	13,4347826	10,77520760	23
		risp680	11,3958333	12,64840527	24
		risp85	11,1500000	10,99600718	24
		Total	10,5821429	10,77433513	140
Total	t1	risp0	2,4583333	1,14473432	6
		risp0.00032	6,5000000	1,14017543	6
		risp170	2,7916667	1,69127664	6
		risp340	5,0000000	1,82537667	6
		risp680	1,9166667	1,07780641	6
		risp85	3,1000000	2,02780670	6
		Total	3,6277778	2,15683116	36
	t2	risp0	23,5833333	6,21624217	6
		risp0.00032	34,6000000	3,62973828	5
		risp170	7,6000000	2,77037904	5
		risp340	27,9166667	7,36489421	6
		risp680	30,8333333	9,85731539	6
		risp85	26,7500000	9,16924206	6
		Total	25,4558824	10,59900485	34
	t3	risp0	2,2000000	1,30384048	5
		risp0.00032	13,7500000	4,66636904	6
		risp170	3,6666667	2,31660671	6
		risp340	16,8000000	1,15108644	5
		risp680	8,1666667	2,54296415	6
		risp85	11,2500000	5,09656747	6
		Total	9,2941176	5,98750928	34
	t4	risp0	5,5833333	3,55551215	6
		risp0.00032	9,4166667	5,07362461	6
		risp170	,4833333	,29439203	6
		risp340	4,5833333	3,13714307	6
		risp680	4,6666667	1,75119007	6
		risp85	3,5000000	1,41421356	6
		Total	4,7055556	3,85621535	36
	Total	risp0	8,7282609	9,77500088	23
		risp0.00032	15,2608696	11,37368331	23
		risp170	3,4630435	3,11933914	23
		risp340	13,4347826	10,77520760	23

Descriptive Statistics[a]

Dependent Variable: cortisol

stress	time	tto	Mean	Std. Deviation	N
		risp680	11,3958333	12,64840527	24
		risp85	11,1500000	10,99600718	24
		Total	10,5821429	10,77433513	140

a. stress = S+

Tests of Between-Subjects Effects[a]

Dependent Variable: cortisol

Source	Type III Sum of Squares	df	Mean Square	F	Sig.
Corrected Model	14034,680[b]	23	610,203	33,685	,000
Intercept	15994,108	1	15994,108	882,931	,000
stress	,000	0	.	.	.
time	10263,885	3	3421,295	188,867	,000
tto	2119,485	5	423,897	23,401	,000
stress * time	,000	0	.	.	.
stress * tto	,000	0	.	.	.
time * tto	1573,807	15	104,920	5,792	,000
stress * time * tto	,000	0	.	.	.
Error	2101,316	116	18,115		
Total	31813,440	140			
Corrected Total	16135,995	139			

a. stress = S+

b. R Squared = ,870 (Adjusted R Squared = ,844)

Estimated Marginal Means

Grand Mean[a]

Dependent Variable: cortisol

Mean	Std. Error	95% Confidence Interval	
		Lower Bound	Upper Bound
10,713	,361	9,999	11,427

a. stress = S+

Univariate Analysis of Variance

[DataSet1] C:\Users\Angelo\Dropbox\UFRGS HOME\ARTIGOS PIATO ET AL\Artigos under construction\UPF\Renan\Dados renan cortisol.sav

time = t1

Between-Subjects Factors[a]

		Value Label	N
groups	R00032S-	R00032S-	6
	R00032S+	R00032S+	6
	R0S-	R0S-	5
	R0S+	R0S+	6
	R170S-	R170S-	6
	R170S+	R170S+	6
	R340S-	R340S-	6
	R340S+	R340S+	6
	R680S-	R680S-	6
	R680S+	R680S+	6
	R85S-	R85S-	6
	R85S+	R85S+	6

a. time = t1

Descriptive Statistics[a]

Dependent Variable: cortisol

groups	Mean	Std. Deviation	N
R00032S-	6,4833333	1,71745936	6
R00032S+	6,5000000	1,14017543	6
R0S-	5,8000000	2,94957624	5
R0S+	2,4583333	1,14473432	6
R170S-	1,7083333	,67854010	6
R170S+	2,7916667	1,69127664	6
R340S-	5,0000000	1,81659021	6
R340S+	5,0000000	1,82537667	6
R680S-	1,9166667	1,12546287	6
R680S+	1,9166667	1,07780641	6
R85S-	3,0833333	2,08366664	6
R85S+	3,1000000	2,02780670	6
Total	3,7852113	2,33824407	71

a. time = t1

Tests of Between-Subjects Effects[a]

Dependent Variable: cortisol

Source	Type III Sum of Squares	df	Mean Square	F	Sig.
Corrected Model	215,942[b]	11	19,631	6,945	,000
Intercept	1029,750	1	1029,750	364,296	,000
groups	215,942	11	19,631	6,945	,000
Error	166,775	59	2,827		
Total	1399,993	71			
Corrected Total	382,717	70			

a. time = t1

b. R Squared = ,564 (Adjusted R Squared = ,483)

Estimated Marginal Means

Grand Mean[a]

Dependent Variable: cortisol

Mean	Std. Error	95% Confidence Interval	
		Lower Bound	Upper Bound
3,813	,200	3,413	4,213

a. time = t1

Post Hoc Tests

groups

Multiple Comparisons[a]

Dependent Variable: cortisol
Bonferroni

(I) groups	(J) groups	Mean Difference (I-J)	Std. Error	Sig.	95% Confidence Interval	
					Lower Bound	Upper Bound
R00032S-	R00032S+	-,0166667	,97068496	1,000	-3,4651498	3,4318165
	R0S-	,6833333	1,01806297	1,000	-2,9334663	4,3001330
	R0S+	4,0250000*	,97068496	,007	,5765168	7,4734832
	R170S-	4,7750000*	,97068496	,000	1,3265168	8,2234832
	R170S+	3,6916667*	,97068496	,023	,2431835	7,1401498
	R340S-	1,4833333	,97068496	1,000	-1,9651498	4,9318165
	R340S+	1,4833333	,97068496	1,000	-1,9651498	4,9318165
	R680S-	4,5666667*	,97068496	,001	1,1181835	8,0151498
	R680S+	4,5666667*	,97068496	,001	1,1181835	8,0151498
	R85S-	3,4000000	,97068496	,058	-,0484832	6,8484832
	R85S+	3,3833333	,97068496	,062	-,0651498	6,8318165
R00032S+	R00032S-	,0166667	,97068496	1,000	-3,4318165	3,4651498
	R0S-	,7000000	1,01806297	1,000	-2,9167997	4,3167997
	R0S+	4,0416667*	,97068496	,007	,5931835	7,4901498
	R170S-	4,7916667*	,97068496	,000	1,3431835	8,2401498
	R170S+	3,7083333*	,97068496	,021	,2598502	7,1568165
	R340S-	1,5000000	,97068496	1,000	-1,9484832	4,9484832
	R340S+	1,5000000	,97068496	1,000	-1,9484832	4,9484832
	R680S-	4,5833333*	,97068496	,001	1,1348502	8,0318165
	R680S+	4,5833333*	,97068496	,001	1,1348502	8,0318165
	R85S-	3,4166667	,97068496	,055	-,0318165	6,8651498
	R85S+	3,4000000	,97068496	,058	-,0484832	6,8484832
R0S-	R00032S-	-,6833333	1,01806297	1,000	-4,3001330	2,9334663
	R00032S+	-,7000000	1,01806297	1,000	-4,3167997	2,9167997
	R0S+	3,3416667	1,01806297	,114	-,2751330	6,9584663
	R170S-	4,0916667*	1,01806297	,011	,4748670	7,7084663
	R170S+	3,0083333	1,01806297	,296	-,6084663	6,6251330
	R340S-	,8000000	1,01806297	1,000	-2,8167997	4,4167997
	R340S+	,8000000	1,01806297	1,000	-2,8167997	4,4167997
	R680S-	3,8833333*	1,01806297	,022	,2665337	7,5001330
	R680S+	3,8833333*	1,01806297	,022	,2665337	7,5001330
	R85S-	2,7166667	1,01806297	,648	-,9001330	6,3334663
	R85S+	2,7000000	1,01806297	,677	-,9167997	6,3167997
R0S+	R00032S-	-4,0250000*	,97068496	,007	-7,4734832	-,5765168
	R00032S+	-4,0416667*	,97068496	,007	-7,4901498	-,5931835
	R0S-	-3,3416667	1,01806297	,114	-6,9584663	,2751330
	R170S-	,7500000	,97068496	1,000	-2,6984832	4,1984832
	R170S+	-,3333333	,97068496	1,000	-3,7818165	3,1151498
	R340S-	-2,5416667	,97068496	,740	-5,9901498	,9068165

Multiple Comparisons[a]

Dependent Variable: cortisol
Bonferroni

(I) groups	(J) groups	Mean Difference (I-J)	Std. Error	Sig.	95% Confidence Interval	
					Lower Bound	Upper Bound
	R340S+	-2,5416667	,97068496	,740	-5,9901498	,9068165
	R680S-	,5416667	,97068496	1,000	-2,9068165	3,9901498
	R680S+	,5416667	,97068496	1,000	-2,9068165	3,9901498
	R85S-	-,6250000	,97068496	1,000	-4,0734832	2,8234832
	R85S+	-,6416667	,97068496	1,000	-4,0901498	2,8068165
R170S-	R00032S-	-4,7750000*	,97068496	,000	-8,2234832	-1,3265168
	R00032S+	-4,7916667*	,97068496	,000	-8,2401498	-1,3431835
	R0S-	-4,0916667*	1,01806297	,011	-7,7084663	-,4748670
	R0S+	-,7500000	,97068496	1,000	-4,1984832	2,6984832
	R170S+	-1,0833333	,97068496	1,000	-4,5318165	2,3651498
	R340S-	-3,2916667	,97068496	,082	-6,7401498	,1568165
	R340S+	-3,2916667	,97068496	,082	-6,7401498	,1568165
	R680S-	-,2083333	,97068496	1,000	-3,6568165	3,2401498
	R680S+	-,2083333	,97068496	1,000	-3,6568165	3,2401498
	R85S-	-1,3750000	,97068496	1,000	-4,8234832	2,0734832
	R85S+	-1,3916667	,97068496	1,000	-4,8401498	2,0568165
R170S+	R00032S-	-3,6916667*	,97068496	,023	-7,1401498	-,2431835
	R00032S+	-3,7083333*	,97068496	,021	-7,1568165	-,2598502
	R0S-	-3,0083333	1,01806297	,296	-6,6251330	,6084663
	R0S+	,3333333	,97068496	1,000	-3,1151498	3,7818165
	R170S-	1,0833333	,97068496	1,000	-2,3651498	4,5318165
	R340S-	-2,2083333	,97068496	1,000	-5,6568165	1,2401498
	R340S+	-2,2083333	,97068496	1,000	-5,6568165	1,2401498
	R680S-	,8750000	,97068496	1,000	-2,5734832	4,3234832
	R680S+	,8750000	,97068496	1,000	-2,5734832	4,3234832
	R85S-	-,2916667	,97068496	1,000	-3,7401498	3,1568165
	R85S+	-,3083333	,97068496	1,000	-3,7568165	3,1401498
R340S-	R00032S-	-1,4833333	,97068496	1,000	-4,9318165	1,9651498
	R00032S+	-1,5000000	,97068496	1,000	-4,9484832	1,9484832
	R0S-	-,8000000	1,01806297	1,000	-4,4167997	2,8167997
	R0S+	2,5416667	,97068496	,740	-,9068165	5,9901498
	R170S-	3,2916667	,97068496	,082	-,1568165	6,7401498
	R170S+	2,2083333	,97068496	1,000	-1,2401498	5,6568165
	R340S+	0E-7	,97068496	1,000	-3,4484832	3,4484832
	R680S-	3,0833333	,97068496	,157	-,3651498	6,5318165
	R680S+	3,0833333	,97068496	,157	-,3651498	6,5318165
	R85S-	1,9166667	,97068496	1,000	-1,5318165	5,3651498
	R85S+	1,9000000	,97068496	1,000	-1,5484832	5,3484832

Multiple Comparisons[a]

Dependent Variable: cortisol
Bonferroni

(I) groups	(J) groups	Mean Difference (I-J)	Std. Error	Sig.	95% Confidence Interval	
					Lower Bound	Upper Bound
R340S+	R00032S-	-1,4833333	,97068496	1,000	-4,9318165	1,9651498
	R00032S+	-1,5000000	,97068496	1,000	-4,9484832	1,9484832
	R0S-	-,8000000	1,01806297	1,000	-4,4167997	2,8167997
	R0S+	2,5416667	,97068496	,740	-,9068165	5,9901498
	R170S-	3,2916667	,97068496	,082	-,1568165	6,7401498
	R170S+	2,2083333	,97068496	1,000	-1,2401498	5,6568165
	R340S-	0E-7	,97068496	1,000	-3,4484832	3,4484832
	R680S-	3,0833333	,97068496	,157	-,3651498	6,5318165
	R680S+	3,0833333	,97068496	,157	-,3651498	6,5318165
	R85S-	1,9166667	,97068496	1,000	-1,5318165	5,3651498
	R85S+	1,9000000	,97068496	1,000	-1,5484832	5,3484832
R680S-	R00032S-	-4,5666667*	,97068496	,001	-8,0151498	-1,1181835
	R00032S+	-4,5833333*	,97068496	,001	-8,0318165	-1,1348502
	R0S-	-3,8833333*	1,01806297	,022	-7,5001330	-,2665337
	R0S+	-,5416667	,97068496	1,000	-3,9901498	2,9068165
	R170S-	,2083333	,97068496	1,000	-3,2401498	3,6568165
	R170S+	-,8750000	,97068496	1,000	-4,3234832	2,5734832
	R340S-	-3,0833333	,97068496	,157	-6,5318165	,3651498
	R340S+	-3,0833333	,97068496	,157	-6,5318165	,3651498
	R680S+	0E-7	,97068496	1,000	-3,4484832	3,4484832
	R85S-	-1,1666667	,97068496	1,000	-4,6151498	2,2818165
	R85S+	-1,1833333	,97068496	1,000	-4,6318165	2,2651498
R680S+	R00032S-	-4,5666667*	,97068496	,001	-8,0151498	-1,1181835
	R00032S+	-4,5833333*	,97068496	,001	-8,0318165	-1,1348502
	R0S-	-3,8833333*	1,01806297	,022	-7,5001330	-,2665337
	R0S+	-,5416667	,97068496	1,000	-3,9901498	2,9068165
	R170S-	,2083333	,97068496	1,000	-3,2401498	3,6568165
	R170S+	-,8750000	,97068496	1,000	-4,3234832	2,5734832
	R340S-	-3,0833333	,97068496	,157	-6,5318165	,3651498
	R340S+	-3,0833333	,97068496	,157	-6,5318165	,3651498
	R680S-	0E-7	,97068496	1,000	-3,4484832	3,4484832
	R85S-	-1,1666667	,97068496	1,000	-4,6151498	2,2818165
	R85S+	-1,1833333	,97068496	1,000	-4,6318165	2,2651498
R85S-	R00032S-	-3,4000000	,97068496	,058	-6,8484832	,0484832
	R00032S+	-3,4166667	,97068496	,055	-6,8651498	,0318165
	R0S-	-2,7166667	1,01806297	,648	-6,3334663	,9001330
	R0S+	,6250000	,97068496	1,000	-2,8234832	4,0734832
	R170S-	1,3750000	,97068496	1,000	-2,0734832	4,8234832
	R170S+	,2916667	,97068496	1,000	-3,1568165	3,7401498

Multiple Comparisons[a]

Dependent Variable: cortisol
Bonferroni

(I) groups	(J) groups	Mean Difference (I-J)	Std. Error	Sig.	95% Confidence Interval	
					Lower Bound	Upper Bound
	R340S-	-1,9166667	,97068496	1,000	-5,3651498	1,5318165
	R340S+	-1,9166667	,97068496	1,000	-5,3651498	1,5318165
	R680S-	1,1666667	,97068496	1,000	-2,2818165	4,6151498
	R680S+	1,1666667	,97068496	1,000	-2,2818165	4,6151498
	R85S+	-,0166667	,97068496	1,000	-3,4651498	3,4318165
R85S+	R00032S-	-3,3833333	,97068496	,062	-6,8318165	,0651498
	R00032S+	-3,4000000	,97068496	,058	-6,8484832	,0484832
	R0S-	-2,7000000	1,01806297	,677	-6,3167997	,9167997
	R0S+	,6416667	,97068496	1,000	-2,8068165	4,0901498
	R170S-	1,3916667	,97068496	1,000	-2,0568165	4,8401498
	R170S+	,3083333	,97068496	1,000	-3,1401498	3,7568165
	R340S-	-1,9000000	,97068496	1,000	-5,3484832	1,5484832
	R340S+	-1,9000000	,97068496	1,000	-5,3484832	1,5484832
	R680S-	1,1833333	,97068496	1,000	-2,2651498	4,6318165
	R680S+	1,1833333	,97068496	1,000	-2,2651498	4,6318165
	R85S-	,0166667	,97068496	1,000	-3,4318165	3,4651498

Based on observed means.
The error term is Mean Square(Error) = 2,827.

*. The mean difference is significant at the ,05 level.

a. time = t1

Homogeneous Subsets

cortisol[a]

Means for groups in homogeneous subsets are displayed.
Based on observed means.
The error term is Mean Square(Error) = 2,827.

a. time = t1

time = t2

Between-Subjects Factors[a]

		Value Label	N
groups	R00032S-	R00032S-	6
	R00032S+	R00032S+	5
	R0S-	R0S-	5
	R0S+	R0S+	6
	R170S-	R170S-	6
	R170S+	R170S+	5
	R340S-	R340S-	6
	R340S+	R340S+	6
	R680S-	R680S-	6
	R680S+	R680S+	6
	R85S-	R85S-	6
	R85S+	R85S+	6

a. time = t2

Descriptive Statistics[a]

Dependent Variable: cortisol

groups	Mean	Std. Deviation	N
R00032S-	2,1250000	,70267347	6
R00032S+	34,6000000	3,62973828	5
R0S-	3,7000000	2,43926218	5
R0S+	23,5833333	6,21624217	6
R170S-	2,3333333	1,15830336	6
R170S+	7,6000000	2,77037904	5
R340S-	6,6666667	,87559504	6
R340S+	27,9166667	7,36489421	6
R680S-	,9208333	,56134140	6
R680S+	30,8333333	9,85731539	6
R85S-	1,9583333	1,16636901	6
R85S+	26,7500000	9,16924206	6
Total	14,0293478	13,62666811	69

a. time = t2

Tests of Between-Subjects Effects[a]

Dependent Variable: cortisol

Source	Type III Sum of Squares	df	Mean Square	F	Sig.
Corrected Model	11127,441[b]	11	1011,586	38,460	,000
Intercept	13598,464	1	13598,464	517,013	,000
groups	11127,441	11	1011,586	38,460	,000
Error	1499,213	57	26,302		
Total	26207,413	69			
Corrected Total	12626,654	68			

a. time = t2

b. R Squared = ,881 (Adjusted R Squared = ,858)

Estimated Marginal Means

Grand Mean[a]

Dependent Variable: cortisol

Mean	Std. Error	95% Confidence Interval	
		Lower Bound	Upper Bound
14,082	,619	12,842	15,322

a. time = t2

Post Hoc Tests

groups

Multiple Comparisons[a]

Dependent Variable: cortisol
Bonferroni

(I) groups	(J) groups	Mean Difference (I-J)	Std. Error	Sig.	95% Confidence Interval Lower Bound	Upper Bound
R00032S-	R00032S+	-32,4750000*	3,10548875	,000	-43,5287952	-21,4212048
	R0S-	-1,5750000	3,10548875	1,000	-12,6287952	9,4787952
	R0S+	-21,4583333*	2,96096734	,000	-31,9977135	-10,9189532
	R170S-	-,2083333	2,96096734	1,000	-10,7477135	10,3310468
	R170S+	-5,4750000	3,10548875	1,000	-16,5287952	5,5787952
	R340S-	-4,5416667	2,96096734	1,000	-15,0810468	5,9977135
	R340S+	-25,7916667*	2,96096734	,000	-36,3310468	-15,2522865
	R680S-	1,2041667	2,96096734	1,000	-9,3352135	11,7435468
	R680S+	-28,7083333*	2,96096734	,000	-39,2477135	-18,1689532
	R85S-	,1666667	2,96096734	1,000	-10,3727135	10,7060468
	R85S+	-24,6250000*	2,96096734	,000	-35,1643802	-14,0856198
R00032S+	R00032S-	32,4750000*	3,10548875	,000	21,4212048	43,5287952
	R0S-	30,9000000*	3,24357721	,000	19,3546875	42,4453125
	R0S+	11,0166667	3,10548875	,052	-,0371285	22,0704618
	R170S-	32,2666667*	3,10548875	,000	21,2128715	43,3204618
	R170S+	27,0000000*	3,24357721	,000	15,4546875	38,5453125
	R340S-	27,9333333*	3,10548875	,000	16,8795382	38,9871285
	R340S+	6,6833333	3,10548875	1,000	-4,3704618	17,7371285
	R680S-	33,6791667*	3,10548875	,000	22,6253715	44,7329618
	R680S+	3,7666667	3,10548875	1,000	-7,2871285	14,8204618
	R85S-	32,6416667*	3,10548875	,000	21,5878715	43,6954618
	R85S+	7,8500000	3,10548875	,942	-3,2037952	18,9037952
R0S-	R00032S-	1,5750000	3,10548875	1,000	-9,4787952	12,6287952
	R00032S+	-30,9000000*	3,24357721	,000	-42,4453125	-19,3546875
	R0S+	-19,8833333*	3,10548875	,000	-30,9371285	-8,8295382
	R170S-	1,3666667	3,10548875	1,000	-9,6871285	12,4204618
	R170S+	-3,9000000	3,24357721	1,000	-15,4453125	7,6453125
	R340S-	-2,9666667	3,10548875	1,000	-14,0204618	8,0871285
	R340S+	-24,2166667*	3,10548875	,000	-35,2704618	-13,1628715
	R680S-	2,7791667	3,10548875	1,000	-8,2746285	13,8329618
	R680S+	-27,1333333*	3,10548875	,000	-38,1871285	-16,0795382
	R85S-	1,7416667	3,10548875	1,000	-9,3121285	12,7954618
	R85S+	-23,0500000*	3,10548875	,000	-34,1037952	-11,9962048
R0S+	R00032S-	21,4583333*	2,96096734	,000	10,9189532	31,9977135
	R00032S+	-11,0166667	3,10548875	,052	-22,0704618	,0371285
	R0S-	19,8833333*	3,10548875	,000	8,8295382	30,9371285
	R170S-	21,2500000*	2,96096734	,000	10,7106198	31,7893802
	R170S+	15,9833333*	3,10548875	,000	4,9295382	27,0371285
	R340S-	16,9166667*	2,96096734	,000	6,3772865	27,4560468

Multiple Comparisons[a]

Dependent Variable: cortisol
Bonferroni

(I) groups	(J) groups	Mean Difference (I-J)	Std. Error	Sig.	95% Confidence Interval	
					Lower Bound	Upper Bound
	R340S+	-4,3333333	2,96096734	1,000	-14,8727135	6,2060468
	R680S-	22,6625000*	2,96096734	,000	12,1231198	33,2018802
	R680S+	-7,2500000	2,96096734	1,000	-17,7893802	3,2893802
	R85S-	21,6250000*	2,96096734	,000	11,0856198	32,1643802
	R85S+	-3,1666667	2,96096734	1,000	-13,7060468	7,3727135
R170S-	R00032S-	,2083333	2,96096734	1,000	-10,3310468	10,7477135
	R00032S+	-32,2666667*	3,10548875	,000	-43,3204618	-21,2128715
	R0S-	-1,3666667	3,10548875	1,000	-12,4204618	9,6871285
	R0S+	-21,2500000*	2,96096734	,000	-31,7893802	-10,7106198
	R170S+	-5,2666667	3,10548875	1,000	-16,3204618	5,7871285
	R340S-	-4,3333333	2,96096734	1,000	-14,8727135	6,2060468
	R340S+	-25,5833333*	2,96096734	,000	-36,1227135	-15,0439532
	R680S-	1,4125000	2,96096734	1,000	-9,1268802	11,9518802
	R680S+	-28,5000000*	2,96096734	,000	-39,0393802	-17,9606198
	R85S-	,3750000	2,96096734	1,000	-10,1643802	10,9143802
	R85S+	-24,4166667*	2,96096734	,000	-34,9560468	-13,8772865
R170S+	R00032S-	5,4750000	3,10548875	1,000	-5,5787952	16,5287952
	R00032S+	-27,0000000*	3,24357721	,000	-38,5453125	-15,4546875
	R0S-	3,9000000	3,24357721	1,000	-7,6453125	15,4453125
	R0S+	-15,9833333*	3,10548875	,000	-27,0371285	-4,9295382
	R170S-	5,2666667	3,10548875	1,000	-5,7871285	16,3204618
	R340S-	,9333333	3,10548875	1,000	-10,1204618	11,9871285
	R340S+	-20,3166667*	3,10548875	,000	-31,3704618	-9,2628715
	R680S-	6,6791667	3,10548875	1,000	-4,3746285	17,7329618
	R680S+	-23,2333333*	3,10548875	,000	-34,2871285	-12,1795382
	R85S-	5,6416667	3,10548875	1,000	-5,4121285	16,6954618
	R85S+	-19,1500000*	3,10548875	,000	-30,2037952	-8,0962048
R340S-	R00032S-	4,5416667	2,96096734	1,000	-5,9977135	15,0810468
	R00032S+	-27,9333333*	3,10548875	,000	-38,9871285	-16,8795382
	R0S-	2,9666667	3,10548875	1,000	-8,0871285	14,0204618
	R0S+	-16,9166667*	2,96096734	,000	-27,4560468	-6,3772865
	R170S-	4,3333333	2,96096734	1,000	-6,2060468	14,8727135
	R170S+	-,9333333	3,10548875	1,000	-11,9871285	10,1204618
	R340S+	-21,2500000*	2,96096734	,000	-31,7893802	-10,7106198
	R680S-	5,7458333	2,96096734	1,000	-4,7935468	16,2852135
	R680S+	-24,1666667*	2,96096734	,000	-34,7060468	-13,6272865
	R85S-	4,7083333	2,96096734	1,000	-5,8310468	15,2477135
	R85S+	-20,0833333*	2,96096734	,000	-30,6227135	-9,5439532

Multiple Comparisons[a]

Dependent Variable: cortisol
Bonferroni

(I) groups	(J) groups	Mean Difference (I-J)	Std. Error	Sig.	95% Confidence Interval Lower Bound	Upper Bound
R340S+	R00032S-	25,7916667*	2,96096734	,000	15,2522865	36,3310468
	R00032S+	-6,6833333	3,10548875	1,000	-17,7371285	4,3704618
	R0S-	24,2166667*	3,10548875	,000	13,1628715	35,2704618
	R0S+	4,3333333	2,96096734	1,000	-6,2060468	14,8727135
	R170S-	25,5833333*	2,96096734	,000	15,0439532	36,1227135
	R170S+	20,3166667*	3,10548875	,000	9,2628715	31,3704618
	R340S-	21,2500000*	2,96096734	,000	10,7106198	31,7893802
	R680S-	26,9958333*	2,96096734	,000	16,4564532	37,5352135
	R680S+	-2,9166667	2,96096734	1,000	-13,4560468	7,6227135
	R85S-	25,9583333*	2,96096734	,000	15,4189532	36,4977135
	R85S+	1,1666667	2,96096734	1,000	-9,3727135	11,7060468
R680S-	R00032S-	-1,2041667	2,96096734	1,000	-11,7435468	9,3352135
	R00032S+	-33,6791667*	3,10548875	,000	-44,7329618	-22,6253715
	R0S-	-2,7791667	3,10548875	1,000	-13,8329618	8,2746285
	R0S+	-22,6625000*	2,96096734	,000	-33,2018802	-12,1231198
	R170S-	-1,4125000	2,96096734	1,000	-11,9518802	9,1268802
	R170S+	-6,6791667	3,10548875	1,000	-17,7329618	4,3746285
	R340S-	-5,7458333	2,96096734	1,000	-16,2852135	4,7935468
	R340S+	-26,9958333*	2,96096734	,000	-37,5352135	-16,4564532
	R680S+	-29,9125000*	2,96096734	,000	-40,4518802	-19,3731198
	R85S-	-1,0375000	2,96096734	1,000	-11,5768802	9,5018802
	R85S+	-25,8291667*	2,96096734	,000	-36,3685468	-15,2897865
R680S+	R00032S-	28,7083333*	2,96096734	,000	18,1689532	39,2477135
	R00032S+	-3,7666667	3,10548875	1,000	-14,8204618	7,2871285
	R0S-	27,1333333*	3,10548875	,000	16,0795382	38,1871285
	R0S+	7,2500000	2,96096734	1,000	-3,2893802	17,7893802
	R170S-	28,5000000*	2,96096734	,000	17,9606198	39,0393802
	R170S+	23,2333333*	3,10548875	,000	12,1795382	34,2871285
	R340S-	24,1666667*	2,96096734	,000	13,6272865	34,7060468
	R340S+	2,9166667	2,96096734	1,000	-7,6227135	13,4560468
	R680S-	29,9125000*	2,96096734	,000	19,3731198	40,4518802
	R85S-	28,8750000*	2,96096734	,000	18,3356198	39,4143802
	R85S+	4,0833333	2,96096734	1,000	-6,4560468	14,6227135
R85S-	R00032S-	-,1666667	2,96096734	1,000	-10,7060468	10,3727135
	R00032S+	-32,6416667*	3,10548875	,000	-43,6954618	-21,5878715
	R0S-	-1,7416667	3,10548875	1,000	-12,7954618	9,3121285
	R0S+	-21,6250000*	2,96096734	,000	-32,1643802	-11,0856198
	R170S-	-,3750000	2,96096734	1,000	-10,9143802	10,1643802
	R170S+	-5,6416667	3,10548875	1,000	-16,6954618	5,4121285

Multiple Comparisons[a]

Dependent Variable: cortisol
Bonferroni

(I) groups	(J) groups	Mean Difference (I-J)	Std. Error	Sig.	95% Confidence Interval	
					Lower Bound	Upper Bound
	R340S-	-4,7083333	2,96096734	1,000	-15,2477135	5,8310468
	R340S+	-25,9583333*	2,96096734	,000	-36,4977135	-15,4189532
	R680S-	1,0375000	2,96096734	1,000	-9,5018802	11,5768802
	R680S+	-28,8750000*	2,96096734	,000	-39,4143802	-18,3356198
	R85S+	-24,7916667*	2,96096734	,000	-35,3310468	-14,2522865
R85S+	R00032S-	24,6250000*	2,96096734	,000	14,0856198	35,1643802
	R00032S+	-7,8500000	3,10548875	,942	-18,9037952	3,2037952
	R0S-	23,0500000*	3,10548875	,000	11,9962048	34,1037952
	R0S+	3,1666667	2,96096734	1,000	-7,3727135	13,7060468
	R170S-	24,4166667*	2,96096734	,000	13,8772865	34,9560468
	R170S+	19,1500000*	3,10548875	,000	8,0962048	30,2037952
	R340S-	20,0833333*	2,96096734	,000	9,5439532	30,6227135
	R340S+	-1,1666667	2,96096734	1,000	-11,7060468	9,3727135
	R680S-	25,8291667*	2,96096734	,000	15,2897865	36,3685468
	R680S+	-4,0833333	2,96096734	1,000	-14,6227135	6,4560468
	R85S-	24,7916667*	2,96096734	,000	14,2522865	35,3310468

Based on observed means.
The error term is Mean Square(Error) = 26,302.

*. The mean difference is significant at the ,05 level.

a. time = t2

Homogeneous Subsets

cortisol[a]

Means for groups in homogeneous subsets are displayed.
Based on observed means.
The error term is Mean Square(Error) = 26,302.

a. time = t2

time = t3

Between-Subjects Factors[a]

		Value Label	N
groups	R00032S-	R00032S-	6
	R00032S+	R00032S+	6
	R0S-	R0S-	6
	R0S+	R0S+	5
	R170S-	R170S-	6
	R170S+	R170S+	6
	R340S-	R340S-	6
	R340S+	R340S+	5
	R680S-	R680S-	6
	R680S+	R680S+	6
	R85S-	R85S-	6
	R85S+	R85S+	6

a. time = t3

Descriptive Statistics[a]

Dependent Variable: cortisol

groups	Mean	Std. Deviation	N
R00032S-	1,9166667	,91742393	6
R00032S+	13,7500000	4,66636904	6
R0S-	2,8416667	2,02741625	6
R0S+	2,2000000	1,30384048	5
R170S-	2,2083333	,24579802	6
R170S+	3,6666667	2,31660671	6
R340S-	3,3750000	4,07967523	6
R340S+	16,8000000	1,15108644	5
R680S-	1,5750000	2,01016169	6
R680S+	8,1666667	2,54296415	6
R85S-	1,0416667	,33229003	6
R85S+	11,2500000	5,09656747	6
Total	5,6250000	5,67690943	70

a. time = t3

Tests of Between-Subjects Effects[a]

Dependent Variable: cortisol

Source	Type III Sum of Squares	df	Mean Square	F	Sig.
Corrected Model	1784,630[b]	11	162,239	21,432	,000
Intercept	2289,819	1	2289,819	302,490	,000
groups	1784,630	11	162,239	21,432	,000
Error	439,054	58	7,570		
Total	4438,528	70			
Corrected Total	2223,684	69			

a. time = t3

b. R Squared = ,803 (Adjusted R Squared = ,765)

Estimated Marginal Means

Grand Mean[a]

Dependent Variable: cortisol

Mean	Std. Error	95% Confidence Interval	
		Lower Bound	Upper Bound
5,733	,330	5,073	6,392

a. time = t3

Post Hoc Tests

groups

Multiple Comparisons[a]

Dependent Variable: cortisol
Bonferroni

(I) groups	(J) groups	Mean Difference (I-J)	Std. Error	Sig.	95% Confidence Interval	
					Lower Bound	Upper Bound
R00032S-	R00032S+	-11,8333333*	1,58848903	,000	-17,4819556	-6,1847111
	R0S-	-,9250000	1,58848903	1,000	-6,5736223	4,7236223
	R0S+	-,2833333	1,66602135	1,000	-6,2076583	5,6409917
	R170S-	-,2916667	1,58848903	1,000	-5,9402889	5,3569556
	R170S+	-1,7500000	1,58848903	1,000	-7,3986223	3,8986223
	R340S-	-1,4583333	1,58848903	1,000	-7,1069556	4,1902889
	R340S+	-14,8833333*	1,66602135	,000	-20,8076583	-8,9590083
	R680S-	,3416667	1,58848903	1,000	-5,3069556	5,9902889
	R680S+	-6,2500000*	1,58848903	,015	-11,8986223	-,6013777
	R85S-	,8750000	1,58848903	1,000	-4,7736223	6,5236223
	R85S+	-9,3333333*	1,58848903	,000	-14,9819556	-3,6847111
R00032S+	R00032S-	11,8333333*	1,58848903	,000	6,1847111	17,4819556
	R0S-	10,9083333*	1,58848903	,000	5,2597111	16,5569556
	R0S+	11,5500000*	1,66602135	,000	5,6256750	17,4743250
	R170S-	11,5416667*	1,58848903	,000	5,8930444	17,1902889
	R170S+	10,0833333*	1,58848903	,000	4,4347111	15,7319556
	R340S-	10,3750000*	1,58848903	,000	4,7263777	16,0236223
	R340S+	-3,0500000	1,66602135	1,000	-8,9743250	2,8743250
	R680S-	12,1750000*	1,58848903	,000	6,5263777	17,8236223
	R680S+	5,5833333	1,58848903	,057	-,0652889	11,2319556
	R85S-	12,7083333*	1,58848903	,000	7,0597111	18,3569556
	R85S+	2,5000000	1,58848903	1,000	-3,1486223	8,1486223
R0S-	R00032S-	,9250000	1,58848903	1,000	-4,7236223	6,5736223
	R00032S+	-10,9083333*	1,58848903	,000	-16,5569556	-5,2597111
	R0S+	,6416667	1,66602135	1,000	-5,2826583	6,5659917
	R170S-	,6333333	1,58848903	1,000	-5,0152889	6,2819556
	R170S+	-,8250000	1,58848903	1,000	-6,4736223	4,8236223
	R340S-	-,5333333	1,58848903	1,000	-6,1819556	5,1152889
	R340S+	-13,9583333*	1,66602135	,000	-19,8826583	-8,0340083
	R680S-	1,2666667	1,58848903	1,000	-4,3819556	6,9152889
	R680S+	-5,3250000	1,58848903	,093	-10,9736223	,3236223
	R85S-	1,8000000	1,58848903	1,000	-3,8486223	7,4486223
	R85S+	-8,4083333*	1,58848903	,000	-14,0569556	-2,7597111
R0S+	R00032S-	,2833333	1,66602135	1,000	-5,6409917	6,2076583
	R00032S+	-11,5500000*	1,66602135	,000	-17,4743250	-5,6256750
	R0S-	-,6416667	1,66602135	1,000	-6,5659917	5,2826583
	R170S-	-,0083333	1,66602135	1,000	-5,9326583	5,9159917
	R170S+	-1,4666667	1,66602135	1,000	-7,3909917	4,4576583
	R340S-	-1,1750000	1,66602135	1,000	-7,0993250	4,7493250

Multiple Comparisons[a]

Dependent Variable: cortisol
Bonferroni

(I) groups	(J) groups	Mean Difference (I-J)	Std. Error	Sig.	95% Confidence Interval Lower Bound	Upper Bound
	R340S+	-14,6000000*	1,74010255	,000	-20,7877557	-8,4122443
	R680S-	,6250000	1,66602135	1,000	-5,2993250	6,5493250
	R680S+	-5,9666667*	1,66602135	,046	-11,8909917	-,0423417
	R85S-	1,1583333	1,66602135	1,000	-4,7659917	7,0826583
	R85S+	-9,0500000*	1,66602135	,000	-14,9743250	-3,1256750
R170S-	R00032S-	,2916667	1,58848903	1,000	-5,3569556	5,9402889
	R00032S+	-11,5416667*	1,58848903	,000	-17,1902889	-5,8930444
	R0S-	-,6333333	1,58848903	1,000	-6,2819556	5,0152889
	R0S+	,0083333	1,66602135	1,000	-5,9159917	5,9326583
	R170S+	-1,4583333	1,58848903	1,000	-7,1069556	4,1902889
	R340S-	-1,1666667	1,58848903	1,000	-6,8152889	4,4819556
	R340S+	-14,5916667*	1,66602135	,000	-20,5159917	-8,6673417
	R680S-	,6333333	1,58848903	1,000	-5,0152889	6,2819556
	R680S+	-5,9583333*	1,58848903	,027	-11,6069556	-,3097111
	R85S-	1,1666667	1,58848903	1,000	-4,4819556	6,8152889
	R85S+	-9,0416667*	1,58848903	,000	-14,6902889	-3,3930444
R170S+	R00032S-	1,7500000	1,58848903	1,000	-3,8986223	7,3986223
	R00032S+	-10,0833333*	1,58848903	,000	-15,7319556	-4,4347111
	R0S-	,8250000	1,58848903	1,000	-4,8236223	6,4736223
	R0S+	1,4666667	1,66602135	1,000	-4,4576583	7,3909917
	R170S-	1,4583333	1,58848903	1,000	-4,1902889	7,1069556
	R340S-	,2916667	1,58848903	1,000	-5,3569556	5,9402889
	R340S+	-13,1333333*	1,66602135	,000	-19,0576583	-7,2090083
	R680S-	2,0916667	1,58848903	1,000	-3,5569556	7,7402889
	R680S+	-4,5000000	1,58848903	,418	-10,1486223	1,1486223
	R85S-	2,6250000	1,58848903	1,000	-3,0236223	8,2736223
	R85S+	-7,5833333*	1,58848903	,001	-13,2319556	-1,9347111
R340S-	R00032S-	1,4583333	1,58848903	1,000	-4,1902889	7,1069556
	R00032S+	-10,3750000*	1,58848903	,000	-16,0236223	-4,7263777
	R0S-	,5333333	1,58848903	1,000	-5,1152889	6,1819556
	R0S+	1,1750000	1,66602135	1,000	-4,7493250	7,0993250
	R170S-	1,1666667	1,58848903	1,000	-4,4819556	6,8152889
	R170S+	-,2916667	1,58848903	1,000	-5,9402889	5,3569556
	R340S+	-13,4250000*	1,66602135	,000	-19,3493250	-7,5006750
	R680S-	1,8000000	1,58848903	1,000	-3,8486223	7,4486223
	R680S+	-4,7916667	1,58848903	,250	-10,4402889	,8569556
	R85S-	2,3333333	1,58848903	1,000	-3,3152889	7,9819556
	R85S+	-7,8750000*	1,58848903	,000	-13,5236223	-2,2263777

Multiple Comparisons[a]

Dependent Variable: cortisol
Bonferroni

(I) groups	(J) groups	Mean Difference (I-J)	Std. Error	Sig.	95% Confidence Interval	
					Lower Bound	Upper Bound
R340S+	R00032S-	14,8833333*	1,66602135	,000	8,9590083	20,8076583
	R00032S+	3,0500000	1,66602135	1,000	-2,8743250	8,9743250
	R0S-	13,9583333*	1,66602135	,000	8,0340083	19,8826583
	R0S+	14,6000000*	1,74010255	,000	8,4122443	20,7877557
	R170S-	14,5916667*	1,66602135	,000	8,6673417	20,5159917
	R170S+	13,1333333*	1,66602135	,000	7,2090083	19,0576583
	R340S-	13,4250000*	1,66602135	,000	7,5006750	19,3493250
	R680S-	15,2250000*	1,66602135	,000	9,3006750	21,1493250
	R680S+	8,6333333*	1,66602135	,000	2,7090083	14,5576583
	R85S-	15,7583333*	1,66602135	,000	9,8340083	21,6826583
	R85S+	5,5500000	1,66602135	,100	-,3743250	11,4743250
R680S-	R00032S-	-,3416667	1,58848903	1,000	-5,9902889	5,3069556
	R00032S+	-12,1750000*	1,58848903	,000	-17,8236223	-6,5263777
	R0S-	-1,2666667	1,58848903	1,000	-6,9152889	4,3819556
	R0S+	-,6250000	1,66602135	1,000	-6,5493250	5,2993250
	R170S-	-,6333333	1,58848903	1,000	-6,2819556	5,0152889
	R170S+	-2,0916667	1,58848903	1,000	-7,7402889	3,5569556
	R340S-	-1,8000000	1,58848903	1,000	-7,4486223	3,8486223
	R340S+	-15,2250000*	1,66602135	,000	-21,1493250	-9,3006750
	R680S+	-6,5916667*	1,58848903	,007	-12,2402889	-,9430444
	R85S-	,5333333	1,58848903	1,000	-5,1152889	6,1819556
	R85S+	-9,6750000*	1,58848903	,000	-15,3236223	-4,0263777
R680S+	R00032S-	6,2500000*	1,58848903	,015	,6013777	11,8986223
	R00032S+	-5,5833333	1,58848903	,057	-11,2319556	,0652889
	R0S-	5,3250000	1,58848903	,093	-,3236223	10,9736223
	R0S+	5,9666667*	1,66602135	,046	,0423417	11,8909917
	R170S-	5,9583333*	1,58848903	,027	,3097111	11,6069556
	R170S+	4,5000000	1,58848903	,418	-1,1486223	10,1486223
	R340S-	4,7916667	1,58848903	,250	-,8569556	10,4402889
	R340S+	-8,6333333*	1,66602135	,000	-14,5576583	-2,7090083
	R680S-	6,5916667*	1,58848903	,007	,9430444	12,2402889
	R85S-	7,1250000*	1,58848903	,002	1,4763777	12,7736223
	R85S+	-3,0833333	1,58848903	1,000	-8,7319556	2,5652889
R85S-	R00032S-	-,8750000	1,58848903	1,000	-6,5236223	4,7736223
	R00032S+	-12,7083333*	1,58848903	,000	-18,3569556	-7,0597111
	R0S-	-1,8000000	1,58848903	1,000	-7,4486223	3,8486223
	R0S+	-1,1583333	1,66602135	1,000	-7,0826583	4,7659917
	R170S-	-1,1666667	1,58848903	1,000	-6,8152889	4,4819556
	R170S+	-2,6250000	1,58848903	1,000	-8,2736223	3,0236223

Multiple Comparisons[a]

Dependent Variable: cortisol
Bonferroni

(I) groups	(J) groups	Mean Difference (I-J)	Std. Error	Sig.	95% Confidence Interval Lower Bound	Upper Bound
	R340S-	-2,3333333	1,58848903	1,000	-7,9819556	3,3152889
	R340S+	-15,7583333*	1,66602135	,000	-21,6826583	-9,8340083
	R680S-	-,5333333	1,58848903	1,000	-6,1819556	5,1152889
	R680S+	-7,1250000*	1,58848903	,002	-12,7736223	-1,4763777
	R85S+	-10,2083333*	1,58848903	,000	-15,8569556	-4,5597111
R85S+	R00032S-	9,3333333*	1,58848903	,000	3,6847111	14,9819556
	R00032S+	-2,5000000	1,58848903	1,000	-8,1486223	3,1486223
	R0S-	8,4083333*	1,58848903	,000	2,7597111	14,0569556
	R0S+	9,0500000*	1,66602135	,000	3,1256750	14,9743250
	R170S-	9,0416667*	1,58848903	,000	3,3930444	14,6902889
	R170S+	7,5833333*	1,58848903	,001	1,9347111	13,2319556
	R340S-	7,8750000*	1,58848903	,000	2,2263777	13,5236223
	R340S+	-5,5500000	1,66602135	,100	-11,4743250	,3743250
	R680S-	9,6750000*	1,58848903	,000	4,0263777	15,3236223
	R680S+	3,0833333	1,58848903	1,000	-2,5652889	8,7319556
	R85S-	10,2083333*	1,58848903	,000	4,5597111	15,8569556

Based on observed means.
The error term is Mean Square(Error) = 7,570.

*. The mean difference is significant at the ,05 level.

a. time = t3

Homogeneous Subsets

cortisol[a]

Means for groups in homogeneous subsets are displayed.
Based on observed means.
The error term is Mean Square(Error) = 7,570.

a. time = t3

time = t4

Between-Subjects Factors[a]

		Value Label	N
groups	R00032S-	R00032S-	6
	R00032S+	R00032S+	6
	R0S-	R0S-	6
	R0S+	R0S+	6
	R170S-	R170S-	6
	R170S+	R170S+	6
	R340S-	R340S-	6
	R340S+	R340S+	6
	R680S-	R680S-	6
	R680S+	R680S+	6
	R85S-	R85S-	5
	R85S+	R85S+	6

a. time = t4

Descriptive Statistics[a]

Dependent Variable: cortisol

groups	Mean	Std. Deviation	N
R00032S-	7,1666667	1,91485422	6
R00032S+	9,4166667	5,07362461	6
R0S-	3,0000000	2,19089023	6
R0S+	5,5833333	3,55551215	6
R170S-	1,0833333	,37638633	6
R170S+	,4833333	,29439203	6
R340S-	,2791667	,15034682	6
R340S+	4,5833333	3,13714307	6
R680S-	,2000000	,08366600	6
R680S+	4,6666667	1,75119007	6
R85S-	1,0020000	,00447214	5
R85S+	3,5000000	1,41421356	6
Total	3,4476761	3,56925631	71

a. time = t4

Tests of Between-Subjects Effects[a]

Dependent Variable: cortisol

Source	Type III Sum of Squares	df	Mean Square	F	Sig.
Corrected Model	581,690[b]	11	52,881	10,062	,000
Intercept	825,290	1	825,290	157,030	,000
groups	581,690	11	52,881	10,062	,000
Error	310,081	59	5,256		
Total	1735,711	71			
Corrected Total	891,771	70			

a. time = t4

b. R Squared = ,652 (Adjusted R Squared = ,587)

Estimated Marginal Means

Grand Mean[a]

Dependent Variable: cortisol

Mean	Std. Error	95% Confidence Interval	
		Lower Bound	Upper Bound
3,414	,272	2,869	3,959

a. time = t4

Post Hoc Tests

groups

Multiple Comparisons[a]

Dependent Variable: cortisol
Bonferroni

(I) groups	(J) groups	Mean Difference (I-J)	Std. Error	Sig.	95% Confidence Interval	
					Lower Bound	Upper Bound
R00032S-	R00032S+	-2,2500000	1,32358321	1,000	-6,9521996	2,4521996
	R0S-	4,1666667	1,32358321	,170	-,5355329	8,8688663
	R0S+	1,5833333	1,32358321	1,000	-3,1188663	6,2855329
	R170S-	6,0833333*	1,32358321	,002	1,3811337	10,7855329
	R170S+	6,6833333*	1,32358321	,000	1,9811337	11,3855329
	R340S-	6,8875000*	1,32358321	,000	2,1853004	11,5896996
	R340S+	2,5833333	1,32358321	1,000	-2,1188663	7,2855329
	R680S-	6,9666667*	1,32358321	,000	2,2644671	11,6688663
	R680S+	2,5000000	1,32358321	1,000	-2,2021996	7,2021996
	R85S-	6,1646667*	1,38818578	,003	1,2329581	11,0963752
	R85S+	3,6666667	1,32358321	,494	-1,0355329	8,3688663
R00032S+	R00032S-	2,2500000	1,32358321	1,000	-2,4521996	6,9521996
	R0S-	6,4166667*	1,32358321	,001	1,7144671	11,1188663
	R0S+	3,8333333	1,32358321	,349	-,8688663	8,5355329
	R170S-	8,3333333*	1,32358321	,000	3,6311337	13,0355329
	R170S+	8,9333333*	1,32358321	,000	4,2311337	13,6355329
	R340S-	9,1375000*	1,32358321	,000	4,4353004	13,8396996
	R340S+	4,8333333*	1,32358321	,037	,1311337	9,5355329
	R680S-	9,2166667*	1,32358321	,000	4,5144671	13,9188663
	R680S+	4,7500000*	1,32358321	,045	,0478004	9,4521996
	R85S-	8,4146667*	1,38818578	,000	3,4829581	13,3463752
	R85S+	5,9166667*	1,32358321	,002	1,2144671	10,6188663
R0S-	R00032S-	-4,1666667	1,32358321	,170	-8,8688663	,5355329
	R00032S+	-6,4166667*	1,32358321	,001	-11,1188663	-1,7144671
	R0S+	-2,5833333	1,32358321	1,000	-7,2855329	2,1188663
	R170S-	1,9166667	1,32358321	1,000	-2,7855329	6,6188663
	R170S+	2,5166667	1,32358321	1,000	-2,1855329	7,2188663
	R340S-	2,7208333	1,32358321	1,000	-1,9813663	7,4230329
	R340S+	-1,5833333	1,32358321	1,000	-6,2855329	3,1188663
	R680S-	2,8000000	1,32358321	1,000	-1,9021996	7,5021996
	R680S+	-1,6666667	1,32358321	1,000	-6,3688663	3,0355329
	R85S-	1,9980000	1,38818578	1,000	-2,9337085	6,9297085
	R85S+	-,5000000	1,32358321	1,000	-5,2021996	4,2021996
R0S+	R00032S-	-1,5833333	1,32358321	1,000	-6,2855329	3,1188663
	R00032S+	-3,8333333	1,32358321	,349	-8,5355329	,8688663
	R0S-	2,5833333	1,32358321	1,000	-2,1188663	7,2855329
	R170S-	4,5000000	1,32358321	,080	-,2021996	9,2021996
	R170S+	5,1000000*	1,32358321	,019	,3978004	9,8021996
	R340S-	5,3041667*	1,32358321	,012	,6019671	10,0063663

Multiple Comparisons[a]

Dependent Variable: cortisol
Bonferroni

(I) groups	(J) groups	Mean Difference (I-J)	Std. Error	Sig.	95% Confidence Interval Lower Bound	Upper Bound
	R340S+	1,0000000	1,32358321	1,000	-3,7021996	5,7021996
	R680S-	5,3833333*	1,32358321	,009	,6811337	10,0855329
	R680S+	,9166667	1,32358321	1,000	-3,7855329	5,6188663
	R85S-	4,5813333	1,38818578	,108	-,3503752	9,5130419
	R85S+	2,0833333	1,32358321	1,000	-2,6188663	6,7855329
R170S-	R00032S-	-6,0833333*	1,32358321	,002	-10,7855329	-1,3811337
	R00032S+	-8,3333333*	1,32358321	,000	-13,0355329	-3,6311337
	R0S-	-1,9166667	1,32358321	1,000	-6,6188663	2,7855329
	R0S+	-4,5000000	1,32358321	,080	-9,2021996	,2021996
	R170S+	,6000000	1,32358321	1,000	-4,1021996	5,3021996
	R340S-	,8041667	1,32358321	1,000	-3,8980329	5,5063663
	R340S+	-3,5000000	1,32358321	,691	-8,2021996	1,2021996
	R680S-	,8833333	1,32358321	1,000	-3,8188663	5,5855329
	R680S+	-3,5833333	1,32358321	,585	-8,2855329	1,1188663
	R85S-	,0813333	1,38818578	1,000	-4,8503752	5,0130419
	R85S+	-2,4166667	1,32358321	1,000	-7,1188663	2,2855329
R170S+	R00032S-	-6,6833333*	1,32358321	,000	-11,3855329	-1,9811337
	R00032S+	-8,9333333*	1,32358321	,000	-13,6355329	-4,2311337
	R0S-	-2,5166667	1,32358321	1,000	-7,2188663	2,1855329
	R0S+	-5,1000000*	1,32358321	,019	-9,8021996	-,3978004
	R170S-	-,6000000	1,32358321	1,000	-5,3021996	4,1021996
	R340S-	,2041667	1,32358321	1,000	-4,4980329	4,9063663
	R340S+	-4,1000000	1,32358321	,197	-8,8021996	,6021996
	R680S-	,2833333	1,32358321	1,000	-4,4188663	4,9855329
	R680S+	-4,1833333	1,32358321	,164	-8,8855329	,5188663
	R85S-	-,5186667	1,38818578	1,000	-5,4503752	4,4130419
	R85S+	-3,0166667	1,32358321	1,000	-7,7188663	1,6855329
R340S-	R00032S-	-6,8875000*	1,32358321	,000	-11,5896996	-2,1853004
	R00032S+	-9,1375000*	1,32358321	,000	-13,8396996	-4,4353004
	R0S-	-2,7208333	1,32358321	1,000	-7,4230329	1,9813663
	R0S+	-5,3041667*	1,32358321	,012	-10,0063663	-,6019671
	R170S-	-,8041667	1,32358321	1,000	-5,5063663	3,8980329
	R170S+	-,2041667	1,32358321	1,000	-4,9063663	4,4980329
	R340S+	-4,3041667	1,32358321	,125	-9,0063663	,3980329
	R680S-	,0791667	1,32358321	1,000	-4,6230329	4,7813663
	R680S+	-4,3875000	1,32358321	,104	-9,0896996	,3146996
	R85S-	-,7228333	1,38818578	1,000	-5,6545419	4,2088752
	R85S+	-3,2208333	1,32358321	1,000	-7,9230329	1,4813663

Multiple Comparisons[a]

Dependent Variable: cortisol
Bonferroni

(I) groups	(J) groups	Mean Difference (I-J)	Std. Error	Sig.	95% Confidence Interval	
					Lower Bound	Upper Bound
R340S+	R00032S-	-2,5833333	1,32358321	1,000	-7,2855329	2,1188663
	R00032S+	-4,8333333*	1,32358321	,037	-9,5355329	-,1311337
	R0S-	1,5833333	1,32358321	1,000	-3,1188663	6,2855329
	R0S+	-1,0000000	1,32358321	1,000	-5,7021996	3,7021996
	R170S-	3,5000000	1,32358321	,691	-1,2021996	8,2021996
	R170S+	4,1000000	1,32358321	,197	-,6021996	8,8021996
	R340S-	4,3041667	1,32358321	,125	-,3980329	9,0063663
	R680S-	4,3833333	1,32358321	,105	-,3188663	9,0855329
	R680S+	-,0833333	1,32358321	1,000	-4,7855329	4,6188663
	R85S-	3,5813333	1,38818578	,818	-1,3503752	8,5130419
	R85S+	1,0833333	1,32358321	1,000	-3,6188663	5,7855329
R680S-	R00032S-	-6,9666667*	1,32358321	,000	-11,6688663	-2,2644671
	R00032S+	-9,2166667*	1,32358321	,000	-13,9188663	-4,5144671
	R0S-	-2,8000000	1,32358321	1,000	-7,5021996	1,9021996
	R0S+	-5,3833333*	1,32358321	,009	-10,0855329	-,6811337
	R170S-	-,8833333	1,32358321	1,000	-5,5855329	3,8188663
	R170S+	-,2833333	1,32358321	1,000	-4,9855329	4,4188663
	R340S-	-,0791667	1,32358321	1,000	-4,7813663	4,6230329
	R340S+	-4,3833333	1,32358321	,105	-9,0855329	,3188663
	R680S+	-4,4666667	1,32358321	,087	-9,1688663	,2355329
	R85S-	-,8020000	1,38818578	1,000	-5,7337085	4,1297085
	R85S+	-3,3000000	1,32358321	1,000	-8,0021996	1,4021996
R680S+	R00032S-	-2,5000000	1,32358321	1,000	-7,2021996	2,2021996
	R00032S+	-4,7500000*	1,32358321	,045	-9,4521996	-,0478004
	R0S-	1,6666667	1,32358321	1,000	-3,0355329	6,3688663
	R0S+	-,9166667	1,32358321	1,000	-5,6188663	3,7855329
	R170S-	3,5833333	1,32358321	,585	-1,1188663	8,2855329
	R170S+	4,1833333	1,32358321	,164	-,5188663	8,8855329
	R340S-	4,3875000	1,32358321	,104	-,3146996	9,0896996
	R340S+	,0833333	1,32358321	1,000	-4,6188663	4,7855329
	R680S-	4,4666667	1,32358321	,087	-,2355329	9,1688663
	R85S-	3,6646667	1,38818578	,699	-1,2670419	8,5963752
	R85S+	1,1666667	1,32358321	1,000	-3,5355329	5,8688663
R85S-	R00032S-	-6,1646667*	1,38818578	,003	-11,0963752	-1,2329581
	R00032S+	-8,4146667*	1,38818578	,000	-13,3463752	-3,4829581
	R0S-	-1,9980000	1,38818578	1,000	-6,9297085	2,9337085
	R0S+	-4,5813333	1,38818578	,108	-9,5130419	,3503752
	R170S-	-,0813333	1,38818578	1,000	-5,0130419	4,8503752
	R170S+	,5186667	1,38818578	1,000	-4,4130419	5,4503752

Multiple Comparisons[a]

Dependent Variable: cortisol
Bonferroni

(I) groups	(J) groups	Mean Difference (I-J)	Std. Error	Sig.	95% Confidence Interval	
					Lower Bound	Upper Bound
	R340S-	,7228333	1,38818578	1,000	-4,2088752	5,6545419
	R340S+	-3,5813333	1,38818578	,818	-8,5130419	1,3503752
	R680S-	,8020000	1,38818578	1,000	-4,1297085	5,7337085
	R680S+	-3,6646667	1,38818578	,699	-8,5963752	1,2670419
	R85S+	-2,4980000	1,38818578	1,000	-7,4297085	2,4337085
R85S+	R00032S-	-3,6666667	1,32358321	,494	-8,3688663	1,0355329
	R00032S+	-5,9166667*	1,32358321	,002	-10,6188663	-1,2144671
	R0S-	,5000000	1,32358321	1,000	-4,2021996	5,2021996
	R0S+	-2,0833333	1,32358321	1,000	-6,7855329	2,6188663
	R170S-	2,4166667	1,32358321	1,000	-2,2855329	7,1188663
	R170S+	3,0166667	1,32358321	1,000	-1,6855329	7,7188663
	R340S-	3,2208333	1,32358321	1,000	-1,4813663	7,9230329
	R340S+	-1,0833333	1,32358321	1,000	-5,7855329	3,6188663
	R680S-	3,3000000	1,32358321	1,000	-1,4021996	8,0021996
	R680S+	-1,1666667	1,32358321	1,000	-5,8688663	3,5355329
	R85S-	2,4980000	1,38818578	1,000	-2,4337085	7,4297085

Based on observed means.
The error term is Mean Square(Error) = 5,256.

*. The mean difference is significant at the ,05 level.

a. time = t4
