# Supplementary material for: Waterborne Risperidone Decreases Stress Response in Zebrafish
Source: PLoS One. 2015 Oct 16;10(10):e0140800. doi: 10.1371/journal.pone.0140800 (PMC4608780; doi:10.1371/journal.pone.0140800)
Supplement: S3 File — Raw data of behavioral tests. (PDF) [file pone.0140800.s003.pdf]

Dados renan behavior.sav

| ... | tto | stress | totaldist | meanspeed | crossings |
|---|---|---|---|---|---|
| 1 | risp 0 | S- | 10,794 | ,036 | 50,000 |
| 2 | risp 0 | S- | 10,250 | ,034 | 168,000 |
| 3 | risp 0 | S- | 9,131 | ,030 | 40,000 |
| 4 | risp 0 | S- | 13,122 | ,044 | 116,000 |
| 5 | risp 0 | S- | 7,722 | ,026 | 56,000 |
| 6 | risp 0 | S- | 10,957 | ,037 | 68,000 |
| 7 | risp 0 | S+ | 9,801 | ,033 | 40,000 |
| 8 | risp 0 | S+ | 13,894 | ,046 | 98,000 |
| 9 | risp 0 | S+ | 26,671 | ,089 | 370,000 |
| 10 | risp 0 | S+ | 6,381 | ,021 | 53,000 |
| 11 | risp 0 | S+ | 6,174 | ,021 | 27,000 |
| 12 | risp 0 | S+ | 3,210 | ,011 | 13,000 |
| 13 | risp 170 | S- | 22,130 | ,074 | 201,000 |
| 14 | risp 170 | S- | 9,400 | ,031 | 54,000 |
| 15 | risp 170 | S- | 9,830 | ,033 | 58,000 |
| 16 | risp 170 | S- | 12,850 | ,043 | 114,000 |
| 17 | risp 170 | S- | 12,450 | ,041 | 79,000 |
| 18 | risp 170 | S- | 10,980 | ,037 | 69,000 |
| 19 | risp 170 | S+ | 12,020 | ,040 | 64,000 |
| 20 | risp 170 | S+ | 13,384 | ,045 | 85,000 |
| 21 | risp 170 | S+ | 8,741 | ,029 | 39,000 |
| 22 | risp 170 | S+ | 6,511 | ,022 | 10,000 |
| 23 | risp 170 | S+ | 13,500 | ,045 | 97,000 |
| 24 | | | . | . | . |

Dados renan behavior.sav

| | turnangle | timebottom | timemiddle | timeupper |
|---|---|---|---|---|
| 1 | 32234,000 | 195,000 | 60,600 | 44,400 |
| 2 | 42157,000 | 61,700 | 100,000 | 138,300 |
| 3 | 32384,000 | 206,200 | 37,300 | 56,500 |
| 4 | 36606,000 | 100,900 | 66,700 | 132,400 |
| 5 | 24609,000 | 138,600 | 72,100 | 89,200 |
| 6 | 43117,000 | 113,100 | 61,300 | 125,600 |
| 7 | 15863,000 | 224,300 | 26,100 | 49,600 |
| 8 | 36895,000 | 38,900 | 73,200 | 188,000 |
| 9 | 74550,000 | 88,000 | 114,100 | 97,900 |
| 10 | 24862,000 | 136,000 | 134,400 | 29,600 |
| 11 | 14999,000 | 254,100 | 14,300 | 31,600 |
| 12 | 23733,000 | 243,000 | 6,400 | 50,600 |
| 13 | 75044,000 | 76,700 | 62,600 | 160,700 |
| 14 | 29256,000 | 159,100 | 61,400 | 79,400 |
| 15 | 33223,000 | 106,900 | 114,500 | 78,600 |
| 16 | 40695,000 | 131,200 | 57,200 | 111,600 |
| 17 | 39769,000 | 75,400 | 48,000 | 176,600 |
| 18 | 32622,000 | 143,600 | 58,200 | 98,200 |
| 19 | 48794,000 | 79,400 | 30,300 | 190,200 |
| 20 | 22456,000 | 128,600 | 67,400 | 103,900 |
| 21 | 19770,000 | 72,900 | 50,200 | 176,900 |
| 22 | 25323,000 | 2,000 | 6,100 | 291,900 |
| 23 | 27745,000 | 36,000 | 85,800 | 178,200 |
| 24 | . | . | . | . |
