# Supplementary material for: Waterborne Risperidone Decreases Stress Response in Zebrafish
Source: PLoS One. 2015 Oct 16;10(10):e0140800. doi: 10.1371/journal.pone.0140800 (PMC4608780; doi:10.1371/journal.pone.0140800)
Supplement: S4 File — Statistics of behavioral data. (PDF) [file pone.0140800.s004.pdf]

Univariate Analysis of Variance

Between-Subjects Factors

		Value Label	N
tto	risp 0	risp 0	12
	risp 170	risp 170	11
stress	S-	S-	12
	S+	S+	11

Descriptive Statistics

Dependent Variable: totaldist

tto	stress	Mean	Std. Deviation	N
risp 0	S-	10,32933	1,824793	6
	S+	11,02183	8,493524	6
	Total	10,67558	5,868156	12
risp 170	S-	12,94000	4,706370	6
	S+	10,83120	3,085676	5
	Total	11,98145	4,012025	11
Total	S-	11,63467	3,666131	12
	S+	10,93518	6,315730	11
	Total	11,30013	4,997900	23

Tests of Between-Subjects Effects

Dependent Variable: totaldist

Source	Type III Sum of Squares	df	Mean Square	F	Sig.
Corrected Model	23,354[a]	3	7,785	,281	,838
Intercept	2908,611	1	2908,611	105,027	,000
tto	8,367	1	8,367	,302	,589
stress	2,866	1	2,866	,103	,751
tto * stress	11,210	1	11,210	,405	,532
Error	526,184	19	27,694		
Total	3486,476	23			
Corrected Total	549,538	22			

a. R Squared = ,042 (Adjusted R Squared = -,109)

Estimated Marginal Means

1. Grand Mean

Dependent Variable: totaldist

Mean	Std. Error	95% Confidence Interval	
		Lower Bound	Upper Bound
11,281	1,101	8,977	13,584

2. tto

Dependent Variable: totaldist

tto	Mean	Std. Error	95% Confidence Interval	
			Lower Bound	Upper Bound
risp 0	10,676	1,519	7,496	13,855
risp 170	11,886	1,593	8,551	15,220

3. stress

Dependent Variable: totaldist

stress	Mean	Std. Error	95% Confidence Interval	
			Lower Bound	Upper Bound
S-	11,635	1,519	8,455	14,814
S+	10,927	1,593	7,592	14,261

4. tto * stress

Dependent Variable: totaldist

tto	stress	Mean	Std. Error	95% Confidence Interval	
				Lower Bound	Upper Bound
risp 0	S-	10,329	2,148	5,833	14,826
	S+	11,022	2,148	6,525	15,519
risp 170	S-	12,940	2,148	8,443	17,437
	S+	10,831	2,353	5,905	15,757

```
UNIANOVA crossings BY tto stress
  /METHOD=SSTYPE(3)
  /INTERCEPT=INCLUDE
  /POSTHOC=tto stress(TUKEY)
  /EMMEANS=TABLES(OVERALL)
  /EMMEANS=TABLES(tto)
  /EMMEANS=TABLES(stress)
  /EMMEANS=TABLES(tto*stress)
  /PRINT=DESCRIPTIVE
  /CRITERIA=ALPHA(.05)
  /DESIGN=tto stress tto*stress.
```

Univariate Analysis of Variance

Between-Subjects Factors

		Value Label	N
tto	risp 0	risp 0	12
	risp 170	risp 170	11
stress	S-	S-	12
	S+	S+	11

Descriptive Statistics

Dependent Variable: crossings

tto	stress	Mean	Std. Deviation	N
risp 0	S-	83,00000	49,408501	6
	S+	100,16667	135,359398	6
	Total	91,58333	97,561597	12
risp 170	S-	95,83333	55,811886	6
	S+	59,00000	35,163902	5
	Total	79,09091	49,214743	11
Total	S-	89,41667	50,699576	12
	S+	81,45455	100,587637	11
	Total	85,60870	76,816629	23

Tests of Between-Subjects Effects

Dependent Variable: crossings

Source	Type III Sum of Squares	df	Mean Square	F	Sig.
Corrected Model	5479,812[a]	3	1826,604	,279	,840
Intercept	163205,714	1	163205,714	24,939	,000
tto	1146,825	1	1146,825	,175	,680
stress	552,540	1	552,540	,084	,775
tto * stress	4165,714	1	4165,714	,637	,435
Error	124337,667	19	6544,088		
Total	298381,000	23			
Corrected Total	129817,478	22			

a. R Squared = ,042 (Adjusted R Squared = -,109)

Estimated Marginal Means

1. Grand Mean

Dependent Variable: crossings

Mean	Std. Error	95% Confidence Interval	
		Lower Bound	Upper Bound
84,500	16,921	49,085	119,915

2. tto

Dependent Variable: crossings

tto	Mean	Std. Error	95% Confidence Interval	
			Lower Bound	Upper Bound
risp 0	91,583	23,353	42,706	140,461
risp 170	77,417	24,492	26,154	128,680

3. stress

Dependent Variable: crossings

stress	Mean	Std. Error	95% Confidence Interval	
			Lower Bound	Upper Bound
S-	89,417	23,353	40,539	138,294
S+	79,583	24,492	28,320	130,846

4. tto * stress

Dependent Variable: crossings

tto	stress	Mean	Std. Error	95% Confidence Interval	
				Lower Bound	Upper Bound
risp 0	S-	83,000	33,025	13,877	152,123
	S+	100,167	33,025	31,044	169,290
risp 170	S-	95,833	33,025	26,710	164,956
	S+	59,000	36,178	-16,721	134,721

```
UNIANOVA meanspeed BY tto stress
  /METHOD=SSTYPE(3)
  /INTERCEPT=INCLUDE
  /POSTHOC=tto stress(TUKEY)
  /EMMEANS=TABLES(OVERALL)
  /EMMEANS=TABLES(tto)
  /EMMEANS=TABLES(stress)
  /EMMEANS=TABLES(tto*stress)
  /PRINT=DESCRIPTIVE
  /CRITERIA=ALPHA(.05)
  /DESIGN=tto stress tto*stress.
```

Univariate Analysis of Variance

Between-Subjects Factors

		Value Label	N
tto	risp 0	risp 0	12
	risp 170	risp 170	11
stress	S-	S-	12
	S+	S+	11

Descriptive Statistics

Dependent Variable: meanspeed

tto	stress	Mean	Std. Deviation	N
risp 0	S-	,03450	,006189	6
	S+	,03683	,028245	6
	Total	,03567	,019532	12
risp 170	S-	,04317	,015779	6
	S+	,03620	,010281	5
	Total	,04000	,013416	11
Total	S-	,03883	,012291	12
	S+	,03655	,021006	11
	Total	,03774	,016658	23

Tests of Between-Subjects Effects

Dependent Variable: meanspeed

Source	Type III Sum of Squares	df	Mean Square	F	Sig.
Corrected Model	,000[a]	3	8,549E-005	,278	,841
Intercept	,032	1	,032	105,409	,000
tto	9,219E-005	1	9,219E-005	,300	,591
stress	3,067E-005	1	3,067E-005	,100	,756
tto * stress	,000	1	,000	,401	,534
Error	,006	19	,000		
Total	,039	23			
Corrected Total	,006	22			

a. R Squared = ,042 (Adjusted R Squared = -,109)

Estimated Marginal Means

1. Grand Mean

Dependent Variable: meanspeed

Mean	Std. Error	95% Confidence Interval	
		Lower Bound	Upper Bound
,038	,004	,030	,045

2. tto

Dependent Variable: meanspeed

tto	Mean	Std. Error	95% Confidence Interval	
			Lower Bound	Upper Bound
risp 0	,036	,005	,025	,046
risp 170	,040	,005	,029	,051

3. stress

Dependent Variable: meanspeed

stress	Mean	Std. Error	95% Confidence Interval	
			Lower Bound	Upper Bound
S-	,039	,005	,028	,049
S+	,037	,005	,025	,048

4. tto * stress

Dependent Variable: meanspeed

tto	stress	Mean	Std. Error	95% Confidence Interval	
				Lower Bound	Upper Bound
risp 0	S-	,035	,007	,020	,049
	S+	,037	,007	,022	,052
risp 170	S-	,043	,007	,028	,058
	S+	,036	,008	,020	,053

```
UNIANOVA turnangle BY tto stress
  /METHOD=SSTYPE(3)
  /INTERCEPT=INCLUDE
  /POSTHOC=tto stress(TUKEY)
  /EMMEANS=TABLES(OVERALL)
  /EMMEANS=TABLES(tto)
  /EMMEANS=TABLES(stress)
  /EMMEANS=TABLES(tto*stress)
  /PRINT=DESCRIPTIVE
  /CRITERIA=ALPHA(.05)
  /DESIGN=tto stress tto*stress.
```

Univariate Analysis of Variance

Between-Subjects Factors

		Value Label	N
tto	risp 0	risp 0	12
	risp 170	risp 170	11
stress	S-	S-	12
	S+	S+	11

Descriptive Statistics

Dependent Variable: turnangle

tto	stress	Mean	Std. Deviation	N
risp 0	S-	35184,50000	6956,298233	6
	S+	31817,00000	22374,06398	6
	Total	33500,75000	15894,43667	12
risp 170	S-	41768,16667	16885,82832	6
	S+	28817,60000	11562,31245	5
	Total	35881,54545	15549,32394	11
Total	S-	38476,33333	12783,65582	12
	S+	30453,63636	17499,36458	11
	Total	34639,39130	15417,38910	23

Tests of Between-Subjects Effects

Dependent Variable: turnangle

Source	Type III Sum of Squares	df	Mean Square	F	Sig.
Corrected Model	523961116[a]	3	174653705,3	,705	,561
Intercept	27043222784	1	27043222784	109,199	,000
tto	18352810,77	1	18352810,77	,074	,788
stress	380398999,6	1	380398999,6	1,536	,230
tto * stress	131193095,3	1	131193095,3	,530	,476
Error	4705348388	19	247649915,1		
Total	32826720392	23			
Corrected Total	5229309503	22			

a. R Squared = ,100 (Adjusted R Squared = -,042)

Estimated Marginal Means

1. Grand Mean

Dependent Variable: turnangle

Mean	Std. Error	95% Confidence Interval	
		Lower Bound	Upper Bound
34396,817	3291,608	27507,402	41286,232

2. tto

Dependent Variable: turnangle

tto	Mean	Std. Error	95% Confidence Interval	
			Lower Bound	Upper Bound
risp 0	33500,750	4542,851	23992,454	43009,046
risp 170	35292,883	4764,582	25320,498	45265,268

3. stress

Dependent Variable: turnangle

stress	Mean	Std. Error	95% Confidence Interval	
			Lower Bound	Upper Bound
S-	38476,333	4542,851	28968,037	47984,629
S+	30317,300	4764,582	20344,915	40289,685

4. tto * stress

Dependent Variable: turnangle

tto	stress	Mean	Std. Error	95% Confidence Interval	
				Lower Bound	Upper Bound
risp 0	S-	35184,500	6424,561	21737,739	48631,261
	S+	31817,000	6424,561	18370,239	45263,761
risp 170	S-	41768,167	6424,561	28321,406	55214,928
	S+	28817,600	7037,754	14087,411	43547,789

```
UNIANOVA timebottom BY tto stress
  /METHOD=SSTYPE(3)
  /INTERCEPT=INCLUDE
  /POSTHOC=tto stress(TUKEY)
  /EMMEANS=TABLES(OVERALL)
  /EMMEANS=TABLES(tto)
  /EMMEANS=TABLES(stress)
  /EMMEANS=TABLES(tto*stress)
  /PRINT=DESCRIPTIVE
  /CRITERIA=ALPHA(.05)
  /DESIGN=tto stress tto*stress.
```

Univariate Analysis of Variance

Between-Subjects Factors

		Value Label	N
tto	risp 0	risp 0	12
	risp 170	risp 170	11
stress	S-	S-	12
	S+	S+	11

Descriptive Statistics

Dependent Variable: timebottom

tto	stress	Mean	Std. Deviation	N
risp 0	S-	135,91667	56,022617	6
	S+	164,05000	89,671729	6
	Total	149,98333	72,783750	12
risp 170	S-	115,48333	34,998481	6
	S+	63,78000	47,742245	5
	Total	91,98182	47,468301	11
Total	S-	125,70000	45,795693	12
	S+	118,47273	87,602843	11
	Total	122,24348	67,457750	23

Tests of Between-Subjects Effects

Dependent Variable: timebottom

Source	Type III Sum of Squares	df	Mean Square	F	Sig.
Corrected Model	28972,537[a]	3	9657,512	2,579	,084
Intercept	328087,704	1	328087,704	87,626	,000
tto	20813,278	1	20813,278	5,559	,029
stress	793,636	1	793,636	,212	,650
tto * stress	9105,562	1	9105,562	2,432	,135
Error	71139,520	19	3744,185		
Total	443811,820	23			
Corrected Total	100112,057	22			

a. R Squared = ,289 (Adjusted R Squared = ,177)

Estimated Marginal Means

1. Grand Mean

Dependent Variable: timebottom

Mean	Std. Error	95% Confidence Interval	
		Lower Bound	Upper Bound
119,808	12,799	93,019	146,596

2. tto

Dependent Variable: timebottom

tto	Mean	Std. Error	95% Confidence Interval	
			Lower Bound	Upper Bound
risp 0	149,983	17,664	113,012	186,954
risp 170	89,632	18,526	50,856	128,407

3. stress

Dependent Variable: timebottom

stress	Mean	Std. Error	95% Confidence Interval	
			Lower Bound	Upper Bound
S-	125,700	17,664	88,729	162,671
S+	113,915	18,526	75,139	152,691

4. tto * stress

Dependent Variable: timebottom

tto	stress	Mean	Std. Error	95% Confidence Interval	
				Lower Bound	Upper Bound
risp 0	S-	135,917	24,981	83,632	188,202
	S+	164,050	24,981	111,765	216,335
risp 170	S-	115,483	24,981	63,198	167,768
	S+	63,780	27,365	6,505	121,055

```
UNIANOVA timemiddle BY tto stress
  /METHOD=SSTYPE(3)
  /INTERCEPT=INCLUDE
  /POSTHOC=tto stress(TUKEY)
  /EMMEANS=TABLES(OVERALL)
  /EMMEANS=TABLES(tto)
  /EMMEANS=TABLES(stress)
  /EMMEANS=TABLES(tto*stress)
  /PRINT=DESCRIPTIVE
  /CRITERIA=ALPHA(.05)
  /DESIGN=tto stress tto*stress.
```

Univariate Analysis of Variance

Between-Subjects Factors

		Value Label	N
tto	risp 0	risp 0	12
	risp 170	risp 170	11
stress	S-	S-	12
	S+	S+	11

Descriptive Statistics

Dependent Variable: timemiddle

tto	stress	Mean	Std. Deviation	N
risp 0	S-	66,33333	20,337027	6
	S+	61,41667	54,286330	6
	Total	63,87500	39,168079	12
risp 170	S-	66,98333	23,838827	6
	S+	47,96000	31,140536	5
	Total	58,33636	27,762106	11
Total	S-	66,65833	21,128802	12
	S+	55,30000	43,712492	11
	Total	61,22609	33,547066	23

Tests of Between-Subjects Effects

Dependent Variable: timemiddle

Source	Type III Sum of Squares	df	Mean Square	F	Sig.
Corrected Model	1235,542[a]	3	411,847	,333	,802
Intercept	84142,934	1	84142,934	67,963	,000
tto	234,301	1	234,301	,189	,668
stress	818,748	1	818,748	,661	,426
tto * stress	284,283	1	284,283	,230	,637
Error	23523,382	19	1238,073		
Total	110977,500	23			
Corrected Total	24758,924	22			

a. R Squared = ,050 (Adjusted R Squared = -,100)

Estimated Marginal Means

1. Grand Mean

Dependent Variable: timemiddle

Mean	Std. Error	95% Confidence Interval	
		Lower Bound	Upper Bound
60,673	7,360	45,269	76,077

2. tto

Dependent Variable: timemiddle

tto	Mean	Std. Error	95% Confidence Interval	
			Lower Bound	Upper Bound
risp 0	63,875	10,157	42,615	85,135
risp 170	57,472	10,653	35,174	79,769

3. stress

Dependent Variable: timemiddle

stress	Mean	Std. Error	95% Confidence Interval	
			Lower Bound	Upper Bound
S-	66,658	10,157	45,399	87,918
S+	54,688	10,653	32,391	76,986

4. tto * stress

Dependent Variable: timemiddle

tto	stress	Mean	Std. Error	95% Confidence Interval	
				Lower Bound	Upper Bound
risp 0	S-	66,333	14,365	36,268	96,399
	S+	61,417	14,365	31,351	91,482
risp 170	S-	66,983	14,365	36,918	97,049
	S+	47,960	15,736	15,025	80,895

```
UNIANOVA timeupper BY tto stress
  /METHOD=SSTYPE(3)
  /INTERCEPT=INCLUDE
  /POSTHOC=tto stress(TUKEY)
  /EMMEANS=TABLES(OVERALL)
  /EMMEANS=TABLES(tto)
  /EMMEANS=TABLES(stress)
  /EMMEANS=TABLES(tto*stress)
  /PRINT=DESCRIPTIVE
  /CRITERIA=ALPHA(.05)
  /DESIGN=tto stress tto*stress.
```

Univariate Analysis of Variance

Between-Subjects Factors

		Value Label	N
tto	risp 0	risp 0	12
	risp 170	risp 170	11
stress	S-	S-	12
	S+	S+	11

Descriptive Statistics

Dependent Variable: timeupper

tto	stress	Mean	Std. Deviation	N
risp 0	S-	97,73333	40,599097	6
	S+	74,55000	60,790517	6
	Total	86,14167	50,750073	12
risp 170	S-	117,51667	41,787961	6
	S+	188,22000	67,253007	5
	Total	149,65455	63,605477	11
Total	S-	107,62500	40,616547	12
	S+	126,21818	84,739623	11
	Total	116,51739	64,645478	23

Tests of Between-Subjects Effects

Dependent Variable: timeupper

Source	Type III Sum of Squares	df	Mean Square	F	Sig.
Corrected Model	38396,928[a]	3	12798,976	4,542	,015
Intercept	326433,029	1	326433,029	115,839	,000
tto	25442,560	1	25442,560	9,029	,007
stress	3225,929	1	3225,929	1,145	,298
tto * stress	12592,437	1	12592,437	4,469	,048
Error	53541,905	19	2817,995		
Total	404193,790	23			
Corrected Total	91938,833	22			

a. R Squared = ,418 (Adjusted R Squared = ,326)

Estimated Marginal Means

1. Grand Mean

Dependent Variable: timeupper

Mean	Std. Error	95% Confidence Interval	
		Lower Bound	Upper Bound
119,505	11,103	96,265	142,745

2. tto

Dependent Variable: timeupper

tto	Mean	Std. Error	95% Confidence Interval	
			Lower Bound	Upper Bound
risp 0	86,142	15,324	54,068	118,216
risp 170	152,868	16,072	119,229	186,508

3. stress

Dependent Variable: timeupper

stress	Mean	Std. Error	95% Confidence Interval	
			Lower Bound	Upper Bound
S-	107,625	15,324	75,551	139,699
S+	131,385	16,072	97,745	165,025

4. tto * stress

Dependent Variable: timeupper

tto	stress	Mean	Std. Error	95% Confidence Interval	
				Lower Bound	Upper Bound
risp 0	S-	97,733	21,672	52,374	143,093
	S+	74,550	21,672	29,190	119,910
risp 170	S-	117,517	21,672	72,157	162,876
	S+	188,220	23,740	138,531	237,909
